# Ligand–Receptor Interactions and Structure–Function Relationships in Off-Target Binding of the β_3_-Adrenergic Agonist Mirabegron to α_1A_-Adrenergic Receptors

**DOI:** 10.3390/ijms25137468

**Published:** 2024-07-07

**Authors:** Ru Huang, Qingfeng Yu, Alexander Tamalunas, Christian G. Stief, Martin Hennenberg

**Affiliations:** 1 The First Affiliated Hospital of Guangzhou Medical University, Guangzhou 510180, China; sysmhr27@hotmail.com (R.H.); yuqf2010@gmail.com (Q.Y.); 2 Department of Urology, LMU University Hospital, LMU Munich, 80539 Munich, Germany; alexander.tamalunas@med.uni-muenchen.de (A.T.);; 3 Urologische Klinik und Poliklinik, Marchioninistr. 15, 81377 Munich, Germany

**Keywords:** mirabegron, storage symptoms, voiding symptoms, overactive bladder (OAB), benign prostatic hyperplasia (BPH), alpha1-adrenoceptor, lower urinary tract symptoms (LUTSs)

## Abstract

The β_3_-adrenoceptor agonist mirabegron is available for the treatment of storage symptoms of overactive bladder, including frequency, urgency, and incontinence. The off-target effects of mirabegron include binding to α_1_-adrenoceptors, which are central in the treatment of voiding symptoms. Here, we examined the structure–function relationships in the binding of mirabegron to a cryo-electron microscopy structure of α_1A_. The binding was simulated by docking mirabegron to a 3D structure of a human α_1A_-adrenoceptor (7YMH) using Autodock Vina. The simulations identified two binding states: slope orientation involving 10 positions and horizontal binding to the receptor surface involving 4 positions. No interactions occurred with positions constituting the α_1A_ binding pocket, including Asp-106, Ser-188, or Phe-312, despite the positioning of the phenylethanolamine moiety in transmembrane regions close to the binding pocket by contact with Phe-288, -289, and Val-107. Contact with the unique positions of α_1A_ included the transmembrane Met-292 during slope binding and exosite Phe-86 during horizontal binding. Exosite binding in slope orientation involved contact of the anilino part, rather than the aminothiazol end, to Ile-178, Ala-103, and Asn-179. In conclusion, contact with Met-292 and Phe-86, which are unique positions of α_1A_, accounts for mirabegron binding to α_1A_. Because of its lack of interactions with the binding pocket, mirabegron has lower affinity compared to α_1A_-blockers and no effects on voiding symptoms.

## 1. Introduction

**Figure 1:**
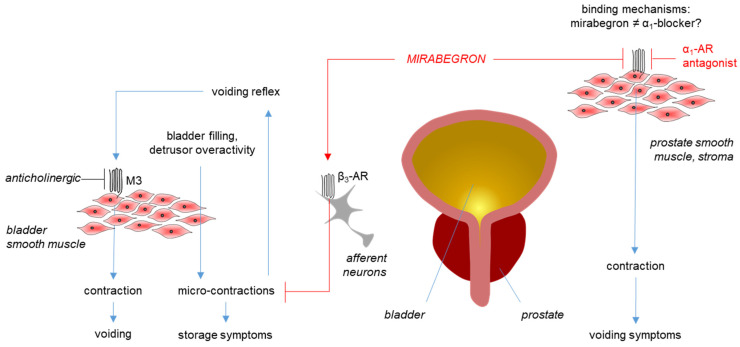
Mirabegron actions in the lower urinary tract. Mirabegron improves storage symptoms in overactive bladder (OAB) by inhibiting afferent signaling by mechanosensitive neurons (which activate the voiding reflex following bladder filling) or by inhibition of microcontractions (which are involved in the activation of the voiding reflex as well). Previous concepts that mirabegron improves storage symptoms by directly inhibiting voiding contractions induced by muscarinic receptors (M3) were not proven. In the prostate, mirabegron antagonizes α_1A_-adrenoceptors (α_1_-AR) but does not improve voiding symptoms, although α_1_-adrenoceptor antagonists (α_1_-blockers) are the gold standard for medical treatment of voiding symptoms in benign prostatic hyperplasia (BPH). The binding of mirabegron to α_1A_-ARs occurs with an affinity of 0.5–5 μM, ranging higher than its maximum plasma levels (167 nM) or than affinities of α_1_-selective adrenoceptor antagonists (low nanomolar ranges). However, the structure–function relationships during mirabegron binding to α_1A_, i.e., the ligand–receptor interactions, and the reasons for its lower affinity to α_1A_ compared to selective ligands are unknown.

Mirabegron is the first β_3_-adrenoceptor agonist approved for the medical treatment of storage symptoms of overactive bladder (OAB) [1,2]. Phase III trials and meta-analyses have confirmed improvements in storage symptoms and incontinence, with an effectiveness similar to that of anticholinergics, which are still the first-line option in the medical treatment of OAB [1,2,3,4,5,6,7,8]. Unlike anticholinergics, which have limiting side effects contributing to discontinuation rates of up to 90%, mirabegron and placebos have comparable rates of adverse events [1,3]. Consequently, and given the high prevalence of OAB, which continuously increases with demographic transitions, β_3_-agonists will gain further relevance in the near future [1,9]. Proposed mechanisms for symptom improvements include the activation of β_3_-adrenergic receptors on bladder smooth muscle cells and on neurons involved in micturition control (Figure 1) [1,10].

Following its approval, the binding of mirabegron to α_1_-adrenoceptors was identified as a major off-target in the prostate and in vascular smooth muscle. In radioligand assays performed in cells transfected with human β-adrenoceptors, the binding constants for β_3_-, β_1_-, and β_2_-adrenoceptors were 2.5 nM, 383 nM, and 977 nM, respectively [11]. In contrast, affinities for α_1_-adrenoceptors in competition assays using membranes from cells transfected with human α_1_-adrenoceptors amounted to 0.437 µM for α_1A_-, 1.8 µM for α_1D_-, and 26 µM for α_1B_-adrenoceptors [12]. The antagonism of α_1_-adrenoceptors was confirmed by functional contraction experiments with intact tissues and required concentrations of 5 µM or more in human prostate tissues [13], and at least 1 µM in prostate, urethral, and aortic rodent tissues (Figure 1) [12]. The predominant subtypes accounting for contractions are α_1A_ in the prostate and urethra [14], and α_1D_ in the rat aorta, while no antagonism was found in rodent spleens, where contractions are caused by α_1B_ [12]. Prostatic α_1_-adrenoceptors are a central target for drug treatment of voiding symptoms in benign prostatic hyperplasia (BPH) (Figure 1), often occurring together with storage symptoms, in men, while vascular α_1_-adrenoceptors account for limiting side effects [14]. Consequently, antagonism of α_1_-adrenoceptors by mirabegron and other β_3_-agonists attracted a specific interest [15]. Meanwhile, binding to α_1_-adrenoceptors has been suggested for a panel of β-adrenergic ligands, including at least 10 drugs used for the treatment of hypertension, overactive bladder, and obstructive lung diseases, thereby imparting an obvious clinical relevance to these off-target effects [16].

The binding of mirabegron to the β_3_-adrenoceptor has been recently simulated by docking approaches using the cryo-electron microscopy (cryo-EM) structure of a β_3_-adrenoceptor [17], but simulations for α_1A_ are not yet available. Cryo-EM structures of human α_1A_-adrenoceptors allowing docking simulation have recently become available for the first time [18,19], while previous simulations were performed using homology-based models for α_1A_, partly combined with site-directed mutagenesis [20,21,22,23]. Across all models, aspartic acid-106 was involved in the binding of all examined α_1_-adrenergic agonists and antagonists to α_1A_. Together with phenylalanine-312 and serine-188, this position constitutes the binding pocket in α_1A_ [18,19,21]. According to the “Eason-Stedman hypothesis”, catecholamine binds to the orthosteric binding pocket due to interactions of the protonated amine with phenylalanine-312, the hydroxyl groups at the chiral center with aspartatic acid-106, and the hydroxyl groups at the aromatic ring of the phenylethanolamine backbone with serine-188 in α_1A_, or with conserved or homologous positions in other subtypes of adrenergic receptors [23,24]. With its chiral center and the amine, phenylethanolamine can still interact with the binding pockets of adrenergic receptors [16]. As the structure of mirabegron is based on a phenylethanolamine backbone, its off-target binding to α_1_-adrenoceptors was initially explained by binding of the phenylethanolamine moiety to the binding pocket [16]. Meanwhile, the concept has been provisionally disproven through the examination of structurally different β_3_-agonists in their ability to antagonize α_1_-adrenergic smooth muscle contractions [15]. In addition to interactions with the orthosteric site, the binding of α_1_-adrenergic antagonists depends on interactions with extracellular receptor regions and on transmembrane residues involved in ligand positioning, including at least two unique positions of α_1A_ [22,23,25].

Studies simulating the off-target binding of mirabegron to the α_1A_-adrenoceptor are not yet available (Figure 1). Here, we report the results from docking simulations using a stabilized cryo-EM structure of α_1A_, aiming to identify possible receptor positions involved in the binding of mirabegron to α_1A_.

## 2. Results

### 2.1. Molecular Docking of Mirabegron

A total of ten repeated simulations, performed under identical conditions, consistently point to two possible binding states of mirabegron to the α_1A_-adrenoceptor, based on specific patterns of ligand orientation and on contacts to receptor positions. Binding occurred either in slope orientation, involving transmembrane regions and exosites, seen in seven out of ten simulations, or in horizontal orientation, involving only surface regions of the receptor, which was present in three of the ten simulations.

**Figure 2:**
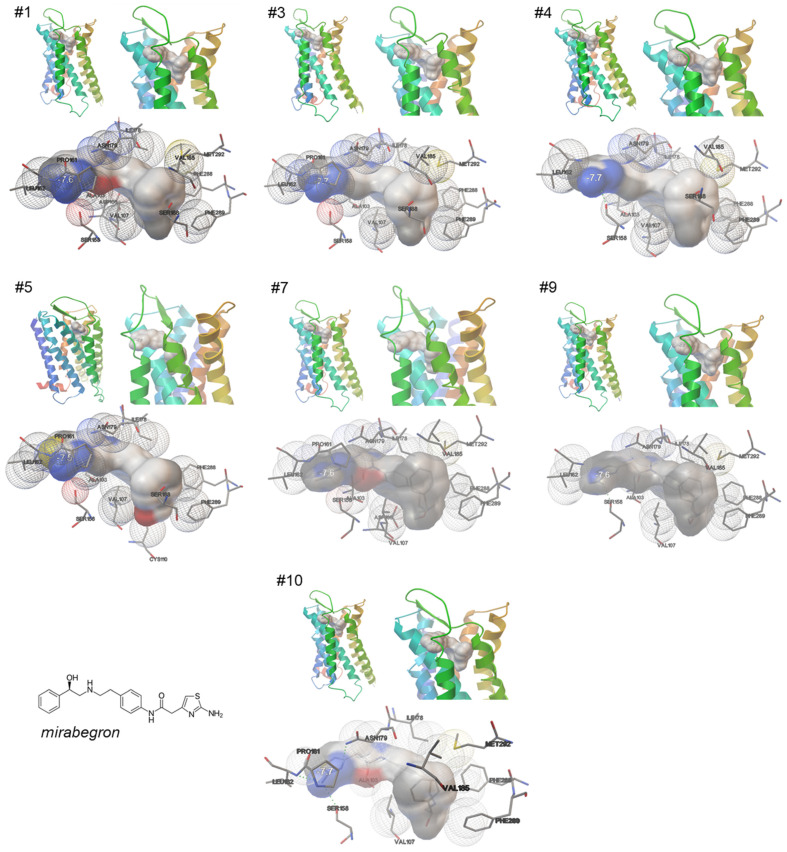
Binding of mirabegron to an active, nanobody-stabilized human α_1A_-adrenoceptor (Nb29-alpha1A AR-miniGsq complex bound to noradrenaline, PDB ID 7YMH), in slope orientation. Shown are 7 of a total of 10 independent simulations, performed under identical conditions (Autodock Vina), with all 7 simulations showing similar results, including binding of mirabegron in slope orientation, and 10 close contacts shared by all 7 simulations (marked by bold font in text boxes). See Supplementary Materials for magnified versions.

In all seven simulations showing slope binding (Figure 2), mirabegron protruded into the transmembrane region, with the phenylethanolamine part ahead and reaching the binding pocket. Both the anilino part and the 2-amino-thiazol end are positioned on surface regions of the receptor, including close contact of the 2-amino-thiazole moiety with the same receptor positions in all seven simulations (Figure 2). The number of close contacts of mirabegron with receptor residues ranged from 10 to 13 in these simulations, with ten of these contacts being seen in each of these seven simulations showing slope binding (Table 1). Shared positions included Ala-103, Val-107, Ser-158, Leu-162, Ile-178, Asn-179, Val-185, Phe-288, Phe-289, and Met-292 (Table 1). Leu-162 and Ser-158 formed close contacts with the 2-amino-thiazol part of mirabegron (Figure 2). The molecule region containing the anilino group formed close contacts with Ala-103 and Asn-179 (Figure 2). Ile-178, still located in or close to surface regions, was in contact with the region between the anilino group and the phenyl end (Figure 2). Val-185, Phe-288, Phe-289, and Met-292 formed close contacts with the aromatic ring of the phenylethanolamine backbone; Val-107 rather formed a close contact with the ethanolamine (Figure 2). At least Val-107, Phe-288, Phe-289, and Met-292 are positioned close to the binding pocket of α_1A_ and account for correct ligand positioning, but close contacts of mirabegron with positions imparting the orthosteric binding, i.e., Asp-106, Phe-312, and Ser-188, were lacking or were only inconsistently observed (Table 1). Contact with Ser-188, formed with the aromatic ring of the phenylethanolamine, was seen in four of the seven simulations with slope binding (Table 1). Contact with Asp-106, formed with the ethanolamine of the phenylethanolamine backbone, was seen in two of the seven slope binding states, while no contact occurred with Phe-312 (Table 1).

**Figure 3:**
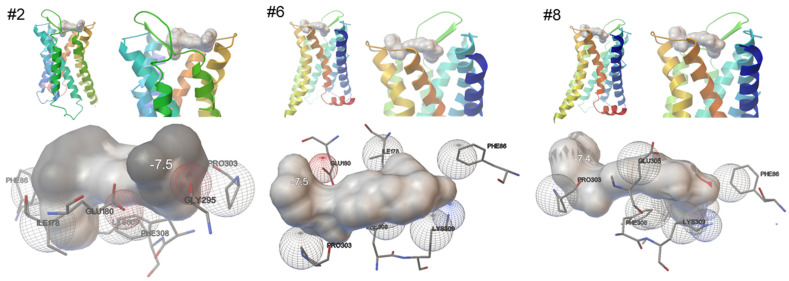
Binding of mirabegron to an active, nanobody-stabilized human α_1A_-adrenoceptor (Nb29-alpha1A AR-miniGsq complex bound to noradrenaline, PDB ID 7YMH), in horizontal orientation. Shown are 3 of a total of 10 independent simulations, performed under identical conditions (Autodock Vina), with all 3 simulations showing similar results, including binding of mirabegron in slope orientation, and 4 close contacts being shared by all 3 simulations (marked by bold font in text boxes). See Supplementary Materials for magnified versions.

In all three simulations showing horizontal binding only to exosites (Figure 3), mirabegron was lying on the top of the receptor, with the 2-amino-thiazol in the center of the receptor’s surface, and the aromatic ring of the phenylethanolamine reaching the extracellular tip of transmembrane helix 6 and the extracellular loop between transmembrane helices 6 and 7. The number of close contacts of mirabegron with receptor residues ranged from five to seven in these simulations, with four of these contacts being seen in each of the seven simulations showing slope binding (Table 1). Shared positions included Phe-86, pro-303, Phe-308, and Lys-309 (Table 1). The phenylethanolamine part formed close contacts with Pro-303, the region between the amino-thiazol and the anilino group, probably by the ketone with Phe-86, and the same molecule region, possibly by the amine with Phe-308 and Phe-309 (Figure 3). Two further positions formed close contacts in two of these three simulations, including Glu-180 forming contact with the ethanolamine, and Ile-178 forming contact with the 2-amino-thiazol end (Figure 3).

### 2.2. Molecular Docking of Tamsulosin

**Figure 4:**
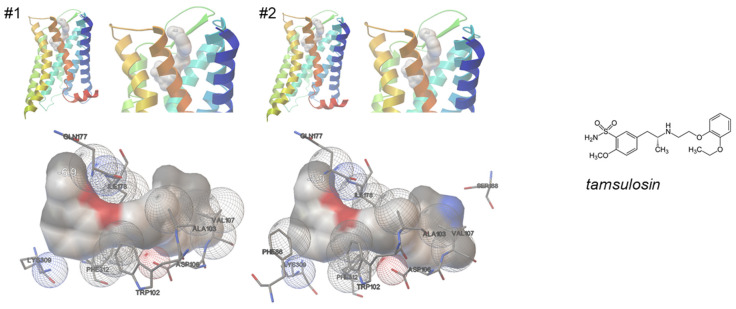
Binding of tamsulosin to an active, nanobody-stabilized human α_1A_-adrenoceptor (Nb29-alpha1A AR-miniGsq complex bound to noradrenaline, PDB ID 7YMH). Shown are both results from 2 independent simulations, performed under identical conditions (Autodock Vina). See Supplementary Materials for magnified versions.

In a total of two repeated simulations, performed under identical conditions, tamsulosin showed nearly identical patterns of binding to the α_1A_-adrenoceptor (Figure 4). In both simulations, tamsulosin bound vertically, with the ethoxyphenoxy group protruding into the binding pocket in the transmembrane region, and the 2-methoxy-benzenesulfonamide in touch with exosites and distal receptor regions (Figure 4).

The number of close contacts of tamsulosin with receptor residues amounted to 8 or 10 in these simulations, with 8 being seen in both simulations (Table 1). Shared positions included Trp-102, Ala-103, Asp-106, Val-107, Gln-177, Ile-178, Lys-309, and Phe-312 (Table 1). Thus, tamsulosin bound to the binding pocket via contacts of the ethyl-aminopropyl backbone to Trp-102, Asp-106, Ile-178, and Phe-312, with Asp-106 and Phe-312 probably via its protonated amine (Figure 4). The ethoxyphenoxy group formed contacts with Ala-103 and Val-107 (Figure 4). The sulfonamide group formed contact with Gln-177, and the 2-methoxybenzene with Lys-309 (Figure 4).

### 2.3. Simulation of Molecular Dynamics in Binding of Mirabegron and Tamsulosin to α_1A_

**Figure 5:**
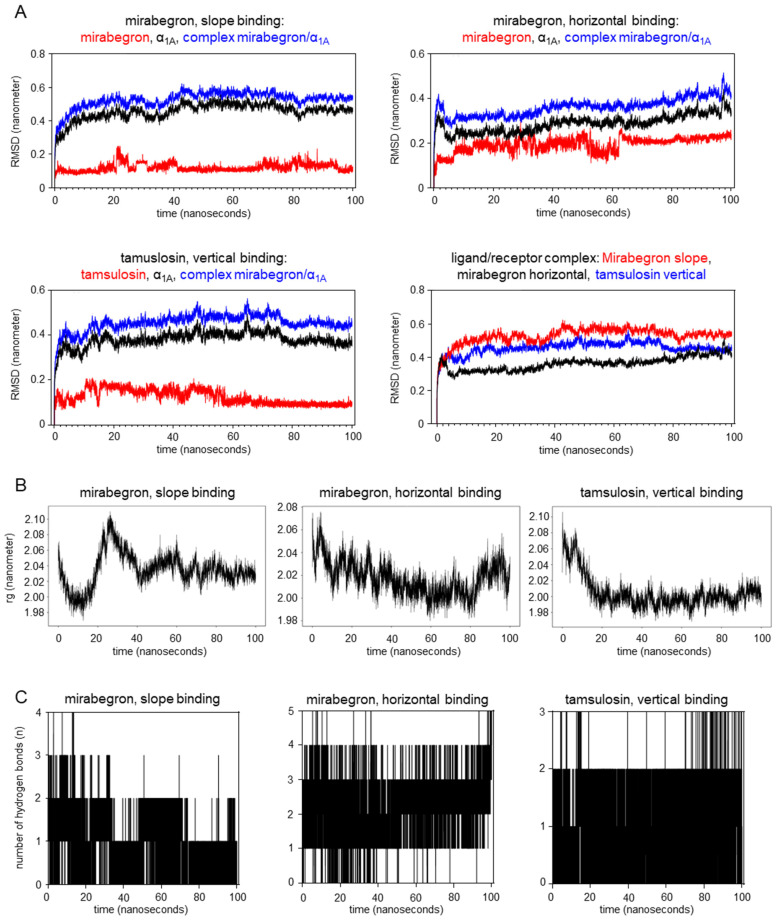
Molecular dynamics in the binding of mirabegron and tamsulosin to the human α_1A_-adrenoceptor. Shown are root mean square deviation (RMSD) of atomic positions (**A**), Gyrate charts (**B**), and numbers of hydrogen bonds (**C**) during the first 100 nanoseconds following slope and horizontal mirabegron binding and vertical tamsulosin binding to human α_1A_-adrenoceptor (PDB ID 7YMH). Simulations were performed using GROMACS. RMSD during the binding process was assessed for ligands and the receptor alone and for ligand–receptor complexes, with alterations and fluctuations in RMSD reflecting conformational changes. The radius of gyration (rg) was assessed for the protein backbone, with changes reflecting changes in the compactness of the ligand–receptor complex and, thus, fluctuations in receptor conformation.

Curves for root mean square deviation (RMSD) of atomic positions from molecular dynamics simulations pointed to fluctuations in receptor conformation in the early stages of binding, occurring with slope and horizontal binding of mirabegron, and with vertical tamsulosin binding to the α_1A_-adrenoceptor (Figure 5A). After 20 nanoseconds, however, the RMSD stabilized with mirabegron and tamsulosin binding to α_1A_, indicating that no further changes in ligand–receptor interactions occurred after fluctuations in initial stages of binding, and that the bindings are stable. Gyrate values after the binding of tamsulosin and after horizontal binding of mirabegron were lower than those of individual protein molecules (Figure 5B), indicating that both bindings increased the protein’s compactness. After the slope binding of mirabegron, Gyrate values fluctuated during the first 40 nanoseconds (Figure 5B), suggesting that the initial binding of mirabegron to α_1A_ caused sequential changes in receptor conformation. Subsequently, i.e., 40 nanoseconds after initial binding in slope orientation, the protein structure became stable again (Figure 5B). Mirabegron and tamsulosin formed hydrogen bonds with the receptor throughout the simulation duration. The number of hydrogen bonds formed with the receptor was highest with horizontal mirabegron binding (Figure 5C). The persistence of hydrogen bond formation over the examined period (0–100 nanoseconds) was higher with horizontal mirabegron binding and tamsulosin binding compared to the slope mirabegron binding (Figure 5C).

**Figure 6:**
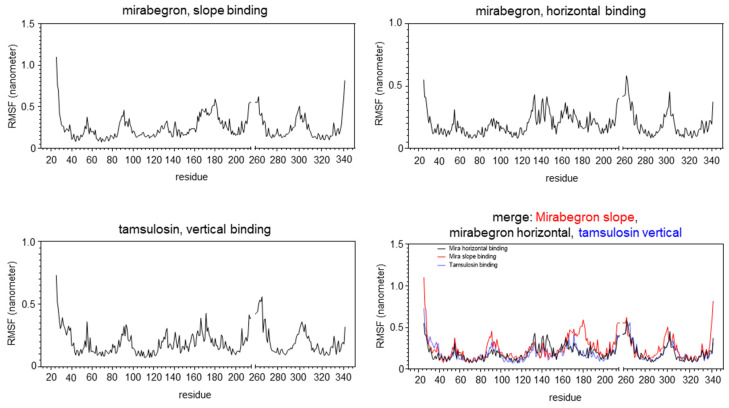
Root mean square fluctuation (RMSF) in the binding of mirabegron and tamsulosin to the human α_1A_-adrenoceptor. RMSF for slope and horizontal mirabegron binding and vertical tamsulosin binding to human α_1A_-adrenoceptor (PDB ID 7YMH) was assessed using GROMACS for the initial 100 nanoseconds of binding. RMSF reflects average deviations of atomic positions from their mean positions during molecular dynamics simulations. Amino acids 215 to 261 in the A chain of α_1A_ are not included in the examined 3D structure of α_1A_ (7YMH), and thus, these residues were not included in the RMSF analysis.

Values for root mean square fluctuation (RMSF) of the protein, calculated for all three bindings (slope and horizontal mirabegron binding, vertical tamsulosin binding), were low in most regions involved in ligand binding and larger in the non-binding regions (Figure 6), indicating that the ligand binding has a certain effect on the conformational stability of α_1A_. However, both binding states of mirabegron showed at least one exception of this pattern, including the region near Glu-180 in slope binding and the region around Asp-300 in horizontal and slope binding. Both regions contain positions interacting with mirabegron in docking simulations, including Ile-178 and Asn-179 in slope binding, Pro-303, Phe-308, and Lys-309 in horizontal binding and Phe-288, -289, and Met-292 in slope binding. These regions showed high RMSF values, different from other regions involved in mirabegron binding and showing lower RMSF values. In contrast, no such deviations were observed for tamsulosin, where all regions involved in binding had low RMSF values.

## 3. Discussion

### 3.1. Two States of Mirabegron Binding May Reflect Dynamic Changes during Binding

Our findings suggest two possible states of mirabegron binding to α_1A_-adrenoceptors, including binding in slope orientation to both transmembrane and surface regions of the receptor and binding in horizontal position only to the receptor surface. Both models showed specific patterns of ligand–receptor contacts. In contrast to our approach, based on a series of repeating simulations, previous studies addressing ligand binding to adrenoceptors mostly reported single states for ligand–receptor interactions or results from a single simulation per study [17,18,19]. Recently, however, simulations for β_2_ revealed that ligand binding includes a sequence of conformational changes, occurring within nanoseconds and altering the binding poses of adrenaline, and with ligand–receptor interactions partly differing between the conformations and depending on ligands and on intracellular receptor coupling [26]. Consequently, dynamic changes during ligand–receptor interactions may theoretically explain our observation of two different states for mirabegron binding to α_1A_, including an early phase with mirabegron docking to the receptor surface by few contacts, followed by steady-state binding involving interior receptor regions and an increased number of contacts. However, molecular dynamic simulations suggested that both binding states stabilize within nanoseconds of initial binding, after fluctuations in receptor conformation with state-specific patterns. Both identified poses probably reflect the binding states with the highest affinities, as they consistently occurred across all ten simulations. Thus, both binding states may occur, resulting in antagonism of α_1A_. As suggested by analysis of hydrogen bonds, however, the stability and affinity are lower when compared with tamsulosin binding, which may account for two possible binding states.

### 3.2. Positions of the Binding Pocket, Imparting Orthosteric Ligand Binding, Are Not Involved in Mirabegron Binding to α_1A_

Several close contacts seen with mirabegron are shared by previous simulations with different α_1_-adrenergic antagonists in different models, while important interactions seen with other ligands in α_1A_ models were lacking in our simulations with mirabegron (Table 2). Previous data are available for the binding of α_1A_-adrenergic ligands in cryo-EM structures of α_1A_ [18,19], and in homology models of α_1A_, developed based on crystal structures of bovine rhodopsin or of a β_2_-adrenoceptor [22,25]. Interaction with Asp-106, seen with all examined α_1_-antagonist and agonists, in all previous simulations [18,19,22,25], was not suggested by our simulations for mirabegron. Asp-106, Phe-312, and Ser-188 constitute the binding pocket of α_1A_ for the orthosteric ligand binding [18,19]. The lack of Asp-106 interaction may explain the lower affinity of mirabegron to α_1A_ compared to α_1A_-selective ligands and the affinity suggested by radioligand binding assays and functional contraction experiments with smooth muscle tissues [12,13]. Consistent with all previous studies using cryo-EM structures or homology models (Table 2), we observed close contacts between Asp-106 and tamsulosin in our simulations, confirming a key role of this position for high-affinity binding to α_1A_ and validating our approach.

Similar to Asp-106, the interaction of Phe-312 was previously reported with noradrenaline, adrenaline, oxymetazoline, and A61603 (the two latter with imidazoline moieties), and with all four examined antagonists in cryo-EM structures and in the β_2_ homology model, but was lacking with mirabegron in our simulations [18,19,25]. Again confirming a key role of this position for high-affinity binding to α_1A_ and validating our approach, we observed close contacts of Phe-312 with tamsulosin in our simulations. The interaction of mirabegron with Ser-188 may not be fully excluded but is probably not decisive, as it was observed in four of seven simulations showing slope binding. In previous simulations, interaction with Ser-188 was found with noradrenaline, adrenaline, oxymetazoline, A61603, tamsulosin, an imidazoline antagonist (“compound 6”), silodosin, RWJ-69736, and SNAP-7915 [18,19,22,25]. Together and different from high-affinity antagonists, the binding of mirabegron to α_1A_ does not involve positions in the binding pocket of α_1A_.

### 3.3. Positions Involved in Slope Binding: Contact with α_1A_-Unique Transmembrane Met-292, and with Exosite Positions

Ten contacts were seen in each of the seven simulations showing slope binding of mirabegron, suggesting that these may be key positions for mirabegron binding to α_1A_. Five of these contacts were repeatedly shared by previous simulations, where they consistently occurred with different α_1_-selective ligands and in different models of α_1A_, including Met-292, Phe-288 and -289, Val-107, and Ile-178 (Table 2). Met-292 is unique for α_1A_, and not conserved in other subtypes of adrenoceptors [18]. Consequently, it seems possible that this interaction accounts for off-target binding of mirabegron to α_1A_. Interactions with met-292 were previously seen with noradrenaline, adrenaline, oxymetazoline, the α_1A_-selective agonist A61603, and tamsulosin in cryo-EM structures [18,19] and with doxazosin and an imidazoline antagonist (N,N0-Bis-(tert-butoxycarbonyl)imidazoline-2-thione) in a homology model of α_1A_ [22]. In line with lacking homologs in other subtypes, Met-292 imparts agonist-selectivity for α_1A_ over α_1B_ [19]. While this position seems decisive for ligand binding to α_1A_, the character of interaction may differ with ligands, including van der Waals interactions with oxymetazoline and tamsulosin, non-polar (not further defined) interaction with noradrenaline [18], and hydrophobic interactions with adrenaline and A61603 [19].

Met-292, Phe-288, -289, and Val-107 are located in transmembrane regions, close to the binding pocket. Accordingly, none of them formed close contacts if mirabegron bound horizontally to the receptor surface. Phe-288, -289, and Val-107 are conserved residues among all adrenoceptors [18]. Although these positions are not involved in orthosteric agonist binding by polar interactions, they are required for correct ligand positioning in the binding pocket, by non-polar and hydrophobic interactions [18,19]. Interactions with Phe-288 and -289 occur with aromatic rings, including those of phenylethanolamine backbones [18,19]. Consequently, they were consistently observed with all examined agonists, including noradrenaline, adrenaline, oxymetazoline, and A61603, and with most examined antagonists, including tamsulosin, silodosin, the imidazoline antagonist, SNAP-7915, and partly with RWJ-69736 in previous simulations with cryo-EM structures and homology models [18,19,22,25]. Provisionally, Val-107 appears less essential for agonist binding but occurred repeatedly in antagonist binding. An interaction was not seen with noradrenaline, but with oxymetazoline, A61603, and tamsulosin in cryo-EM structures, and with silodosin, RWJ-69736, and SNAP-7915 in the β_2_ homology model [18,19,25]. Together, we assume that contact with the α_1A_-unique met-292 enables mirabegron binding to α_1A_, paralleled by positioning in transmembrane regions by contacts of the phenylethanolamine part with Phe-288, -289, and Val-107.

Different from these transmembrane residues, Ile-178 is located at the receptor surface. Accordingly, we observed close contacts to mirabegron in two, though not all, simulations with horizontal surface binding of mirabegron as well, in addition to all simulations showing vertical binding. The binding pocket of α_1A_ is entirely located in transmembrane regions, so previous simulations did not suggest interactions of Ile-178 with most agonists, including noradrenaline, adrenaline, and oxymetazoline [18,19]. An exception is A61603, being larger in molecule size and reaching exosites of the receptor, allowing hydrophobic interaction with Ile-178 [19]. However, close contact between Ile-178 and both mirabegron and tamsulosin suggested by our simulations is consistent with previously reported interactions between Ile-178 and tamsulosin in the rhodopsin-based homology model of [22], and of silodosin and SNAP-7915 in the β_2_ homology model [25]. We assume that the interaction of mirabegron with Ile-178 imparts binding to the receptor surface, in both the slope and horizontal binding states. The interaction appears to occur with the anilino group or its adjacent regions, positioned close to the receptor surface, but not with the 2-amino-thiazol end, while the involvement of the phenylethanolamine part (positioned on transmembrane regions) seems most unlikely. In addition to Ile-178, the anilino group of mirabegron formed close contacts with two exosites, Ala-103 and Asn-179. These positions are not shared by previous simulations with α_1_-selective ligands. It seems possible that binding to exosites of α_1A_ is predominant with mirabegron, compared to α_1_-selective ligands, which may explain the slope orientation of mirabegron but vertical binding of α_1_-antagonists.

### 3.4. Positions Involved in Horizontal Binding: Interaction with α_1A_-Unique Phe-86

In all three simulations showing mirabegron bound only to the receptor surface, in a horizontal orientation and without protruding into transmembrane regions, close contacts occurred with Phe-86, Pro-303, Phe-308, and Lys-309. According to their location at exosites and to the location of the binding pocket to transmembrane regions, none of these positions interacted with α_1_-agonists in previous simulations [18,19]. However, interactions of α_1_-selective antagonists were reported with Phe-308 and Phe-86 (Table 2). Phe-308 formed non-polar interactions with the sulfonamide group of tamsulosin in a cryo-EM structure [18], hydrophobic interactions with silodosin, RWJ-69736, and SNAP-7915 in the β_2_-based homology model [25], and again hydrophobic interactions with an imidazoline antagonist in the rhodopsin homology model [22]. A key role in antagonist binding has been recently assigned to Phe-86, which is unique for α_1A_ and not conserved in other subtypes of adrenoceptors [18]. The methoxybenzene group of tamsulosin formed non-polar interactions with Phe-86 in simulations using a cryo-EM structure, confirming previous mutagenesis studies identifying the position as a determinant for binding of prazosin and HEAT to α_1A_ [18]. We assume that interaction with Phe-86 holds a key role in mirabegron binding to α_1A_ as well, specifically during the first contact, while other positions become decisive in later stages of binding, including the α_1A_-unique Met-292 during steady-state binding.

### 3.5. Mirabegron Binding to the Orthosteric Binding Pocket of β_3_

The binding of mirabegron to β_3_ has been recently investigated using a cryo-EM structure of a β_3_-adrenoceptor in complex with Gαs [17]. The binding of mirabegron to β_3_ was mostly imparted by interactions of the chiral hydroxyl group and of the amine of the phenylethanolamine core to the orthosteric ligand binding pocket [17]. Both the chiral hydroxyl and the amine interacted with Asn-332, while the hydroxyl group additionally interacted with Asp-117 [17]. Mirabegron fitted vertically to the binding pocket, with the phenylethanolamine moiety docking to the orthosteric site, while the 2-amino-thiazole tail additionally interacted with an extracellular receptor site [17]. In total, 17 receptor positions including hydrophobic interactions are involved in the binding of mirabegron to β_3_ in this simulation [17]. Analogously to the mirabegron interaction with Asp-117 in β_3_ [19], all previously examined α_1_-selective ligands interacted with the homolog Asp-106 in the binding pocket of α_1A_ (Table 2). The interaction often included amines and nitrogens, or their adjacent molecule regions. Interactions were imparted by a H-bond with the central ethylamine of tamsulosin, the chiral hydroxyl group of noradrenaline [18], electrostatic interaction with the protonated amine of silodosin, but also with the nitrogen in the indole part of silodosin, by ionic interaction with the protonated amine in the piperazine group of RWJ-69736, and with the protonated nitrogen of the piperidine ring in SNAP-7915 [25]. As mirabegron contains three protonated nitrogens [16], an interaction with Asp-106 in α_1A_ may be expected as well, but it did not consistently occur in our simulations. Steric arrangements in the binding pocket differ in the binding of mirabegron and α_1_-selective ligands to α_1A_, or between arrangements in mirabegron binding to α_1A_ and β_3_, preventing sufficient contact between mirabegron and Asp-106 in α_1A_ but allowing the contact with Asp-117 in β_3_. The mirabegron-specific arrangement may result from conformational changes caused by interactions seen with mirabegron but not occurring with α_1_-specific ligands. We observed the binding of mirabegron in slope orientation, while α_1_-selective antagonists mostly bound vertically.

### 3.6. Molecular Dynamics of Mirabegron and Tamsulosin Binding to α_1A_

Since proteins and small ligands can be in absolute motion, conformations involved in molecular docking may not necessarily represent stationary states [27,28,29]. In fact, activation of G protein-coupled receptors by endogenous ligands and binding of synthetic ligands results in conformational changes of receptors [27,28,29]. Sequences of conformational changes may occur within nanoseconds, from initial to steady-state ligand binding, as recently shown for the β_2_-adrenoceptor [26]. Conformational changes were suggested by RMSD curves in our molecular dynamics studies, with high fluctuations in the initial stages of horizontal mirabegron, before the binding becomes stable within 20 nanoseconds and to a lower degree for slope binding of mirabegron and vertical binding of tamsulosin, both becoming stable within 15 nanoseconds or earlier. Fluctuations in receptor conformation during initial binding phases were confirmed by Gyrate values. Gyrate values highly fluctuated during the initial 20–40 nanoseconds of slope mirabegron binding and of tamsulosin binding, before becoming stable, and across the whole analyzed period of horizontal mirabegron binding, reflecting dynamics in compactness of the receptor protein and, thus, dynamic conformational changes within these periods.

Our findings from hydrogen bond analyses together with RMFS charts may suggest that the binding of mirabegron to α_1A_ occurs with lower affinity and could be less stable than the binding of tamsulosin. Compared to tamsulosin, mirabegron formed fewer hydrogen bonds, at least in slope binding, while the persistence of hydrogen bonds was highest and most stable with tamsulosin. In line, RMSF values were highly consistent with binding and non-binding regions of α_1A_ in tamsulosin binding, while slope and horizontal mirabegron binding each included at least one binding region deviating from this consistency. Thus, these findings may confirm previous in vitro findings, suggesting antagonism of α_1A_ by mirabegron, occurring with lower affinity compared to α_1A_-selective antagonists such as tamsulosin [12,13]. Interestingly, the two regions deviating in RMFS analyses are positioned close to each other within the receptor conformation, confirming that this receptor site is decisive for both binding states. Of note, RMFS deviation was shared by the horizontal and slope binding for the region around Asp-300, which includes positions showing interactions with mirabegron in both binding states. Thus, horizontal and slope binding show distinct, state-specific patterns and interactions, but also shared mechanisms and interactions during their binding to α_1A_. The shared (or any other) RMSF deviation was lacking or less pronounced for tamsulosin binding, suggesting that interactions of mirabegron with positions in this region are characteristic for or impart the unspecific binding of mirabegron.

### 3.7. Clinical Aspects

Mirabegron is available for the treatment of female and male storage symptoms in OAB [1,2]. In men, OAB and storage symptoms often occur together with voiding symptoms in BPH, where α_1A_-adrenergic contractions and prostate smooth muscle contraction are critical in the etiology and treatment of symptoms, and α_1_-blockers represent the first-line option of medical treatment [30]. In in vitro contraction experiments with human prostate tissues, antagonism in concentration–response curves for α_1_-adrenergic agonists started with mirabegron concentrations of 5 µM, but was lacking with 1 µM [13]. In contrast, peak plasma levels during standard dosing of 50 mg/d do not exceed 167 nM, and steady-state plasma concentrations mostly range below 100 nM [31]. Safety and plasma levels have been examined up to 300 mg/d, resulting in peak plasma levels of 961 nM in men, but not being clinically available [30,31].

Antagonism of α_1_-adrenoceptors in preclinical studies initiated clinical studies, with the hope that mirabegron may improve voiding symptoms suggestive of BPH as well, in parallel to storage symptoms. However, according to its affinity for α_1A_ exceeding maximum plasma levels, monotherapy with mirabegron did not improve voiding symptoms in prospective trials [32,33]. Similarly, improvements by add-on to α_1_-blockers were small and probably out of clinical relevance, or lacking [34,35,36]. Nonetheless, structure–function relationships, i.e., the possible ligand–receptor interactions during mirabegron binding to α_1A_, subsequently attracted attention [16]. Initial speculations that mirabegron and other β_3_-adrenergic ligands bind to α_1A_ by interactions between catecholamine backbones with the orthosteric binding pocket of α_1A_ did not prove true [16], as it was subsequently observed that structurally different β_3_-agonists without catecholamine moieties may antagonize α_1A_ as well [15]. Thus, previous preclinical studies did not explain the lower affinity of mirabegron to α_1A_, compared to α_1_-selective antagonists, or the lack of effects on voiding symptoms despite antagonism of α_1A_. Consequently, our current study completes previous gaps between preclinical and clinical studies and allows an understanding of discrepant findings in vitro and in vivo. Specifically, an interaction with Asp-106 appears essential for the high-affinity binding of ligands to α_1A_-adrenoceptors and, thus, for the improvement in voiding symptoms in BPH, which is lacking with mirabegron. Despite its clinical application, mirabegron’s actions are insufficiently understood, even including mechanisms underlying improvements in storage symptoms and raising an obvious need for ongoing investigations [10,37]. From a visionary view, understanding the structure–function relationships underlying mirabegron binding to α_1A_-adrenoceptors could be helpful for the directed development of drugs with dual activities as β_3_-agonists and α_1A_-antagonists to avoid polypharmacy by simultaneous treatment of voiding and storage symptoms by a single drug in future, which currently requires combination therapies with low tolerability.

### 3.8. Limitations

In attempts to validate our approach, we simulated the docking of tamsulosin to an active α_1A_-adrenoceptor, resulting in large concordance with previous findings. Four out of the eight interactions seen in both of our simulations with tamsulosin were previously reported from a nanobody-stabilized, cryo-EM structure as well (7YMJ), including Asp-106, Trp-106, Val-107, and Phe-312, and two were shared by findings from the rhodopsin-based homology model, including Asp-106 and Ile-178. The structure used for our simulations represents an active, noradrenaline-bound state of the receptor, while previous antagonist docking to a cryo-EM structure represented the inactive, i.e., tamsulosin-bound, receptor. Docking of mirabegron to an active receptor may not necessarily represent a true limitation, as this may resemble conditions in vivo or radioligand competition assays, even though antagonist binding to unoccupied receptors occurs of course as well. The comparability of findings from the latest 3D structures, including ours, to homology models could be limited, as some calculated positions naturally deviate from the true α_1A_-adrenoceptor. Different from some, though not all, previous simulations addressing ligand binding to α_1A_, our simulations simply point to close contacts between receptor positions and ligands, but not to the character of possible interactions. The applied program predicts binding modes, binding sites, and similar aspects of a protein–ligand complex by analyzing potential interactions. However, interactions are mostly indicated as close contacts, not allowing reliable analysis of the types of interactions themselves. Thus, follow-up studies may attempt to identify the character of interactions using alternative in silico methods or may include mutagenesis studies to confirm the relevance of identified positions in mirabegron binding to α_1A_. Nevertheless, non-hydrophobic interactions, imparting binding of agonists and α_1_-blockers to positions in the binding pocket (including H-bonds, and electrostatic and ionic interactions) appear not involved in mirabegron binding to α_1A_. Overall, docking simulations may be afflicted by limitations, so overestimation of this approach and too high confidence have been discouraged [38,39]. We performed ten repeated simulations under identical conditions, which provided some different results, but surprisingly high consistencies as well. While our findings explain (a) previous findings from preclinical studies and (b) why this off-target binding does not occur in clinical settings, despite findings from previous preclinical studies, our findings are limited to an in silico approach and need to be confirmed by site-directed mutagenesis studies. Finally, amino acids 215 to 261 in the A chain of α_1A_ are not included in the 3D structure of α_1A_ used here (7YMH), which has been used for molecular docking. Therefore, residue numbers from 215 to 261 have not been included in the RMSF results.

### 3.9. Conclusions

Repeated simulations under the same conditions identify two binding states of mirabegron at α_1A_-adrenoceptors. In the presumed steady state, mirabegron binds in slope orientation, with the phenylethanolamine moiety binding to transmembrane regions, and the anilino group and 2-amino-thiazol end in contact with exosites at the surface, together involving 10 contacts. In the presumed early stage, mirabegron binds horizontally, exclusively to the receptor surface, involving four contacts. Contacts with the α_1A_-unique positions Met-292 in slope binding and Phe-86 in horizontal binding may enable mirabegron binding to transmembrane regions and exosites of α_1A_, while the lack of interaction with the binding pocket, in particular Asp-106, may explain its low affinity compared to α_1A_-selective ligands. Further positions involved in transmembrane positioning include Phe-288, -289, and Val-107, imparting positioning but not direct binding to the binding pocket. Binding to exosites of α_1A_ may predominate with mirabegron compared to α_1_-selective antagonists, as it included Ile-178, reported with α_1_-selective antagonists as well, but also Ala-103 and Asn-179. In particular, interaction with Asp-106 appears essential for high-affinity binding of ligands to α_1A_, which is lacking with mirabegron, explaining the lack of improvements in voiding symptoms in clinical trials with mirabegron.

## 4. Methods

### 4.1. Docking Simulations

Binding simulations were performed using Autodock Vina [40,41]. Docking of mirabegron (CID 5362376) and tamsulosin hydrochloride (CID 5362376) to the human α_1A_-adrenoceptor was performed using the 3D structure of Nb29-alpha1A AR-miniGsq complex bound to noradrenaline (PDB ID 7YMH), which was downloaded from the RCSB Protein Data Bank (RCSB PDB) (www.rcsb.org). The structure represents a 3D, cryo-EM structure of a human α_1A_-adrenoceptor, stabilized by a nanobody, in its active, noradrenaline-bound state. Structure files for mirabegron and tamsulosin were acquired from PubChem and subsequently converted into PDBQT files, using the OpenBabel free software (The Open Babel Package, version 3.1.1 http://openbabel.org).

All docking processes were consistently handled by the same individual. Simulations by the program work widely autonomously. All outcomes with the highest affinity in a single simulation are included in the final results for the given simulation. The docking process in AutoDock Vina depends on random seeds, which are automatically selected by the software itself once the docking procedure begins and ensures the reproducibility of results, by governing the generation of random numbers utilized in various algorithmic steps. These random seeds differ between simulations and cannot be adapted or changed. A total of 10 simulations were run with mirabegron, and 2 simulations were run with tamsulosin. The van der Waals (VDW) scaling factor was set to 1.00 in each simulation.

Autodock Vina is a software for molecular docking, depicting interactions as close contacts, but typically not including details on the type of interactions [40,41]. The program can predict binding modes and energies between small molecules and protein targets by analyzing potential interactions [40,41], but it cannot directly analyze the types of interactions themselves. Each type of interaction is weighted accordingly during the calculation of ligand binding, without the details of these interactions being fully visualized or indicated by Autodock Vina and its accompanying visualization software (Autodock Tools) [40,41]. While hydrogen bonds may be indicated (though not consistently), this does not apply for other interactions, and it is generally not advisable to determine types of interactions relying solely on Autodock Vina, but the focus lies on information on overall binding energies, binding modes, predicted binding sites, and similar aspects of a protein–ligand complex.

### 4.2. Molecular Dynamics Simulations

Dynamics and binding stabilities during interactions between mirabegron and tamsulosin with α_1A_-adrenergic receptors (PDB ID 7YMH) were explored by dynamics simulations, allowing the tracking of protein motions by monitoring their conformational changes over time [42,43,44]. Analyses were performed using GROMACS, providing curves for the root mean square (RMSD) for atomic positions, Gyrate charts as an index for the overall compactness of the protein, root mean square fluctuation (RMSF) reflecting the average deviation of atomic positions from their mean positions during molecular dynamic simulations, and the number of hydrogen bonds over time [42,43,44]. The protein implemented the Amber99sb-ildn force field parameter, while ligand atomic charges were determined using the B3LYP/6-31G* basis set, and ligand topology was constructed using the GAFF2 force field parameter. The TIP3P water model was employed, supplemented with 0.15M (0.9%) NaCl, and the charge was neutralized. Initially, the system underwent energy minimization via the steepest descent method, followed by equilibration in the canonical ensemble (NVT) and the isothermal–isobaric ensemble (NPT), each for 100,000 steps. Coupling constants for both ensembles were set to 0.1 ps, with each equilibration step lasting 100 ps. Subsequently, the molecular dynamics simulation ran for 5,000,000 steps at a constant temperature of 300 K and pressure of 1 bar, with a time step of 2 fs, totaling 100 ns of simulation time.

### 4.3. Ligands

Mirabegron (2-(2-amino-1,3-thiazol-4-yl)-N-[4-[2-[[(2R)-2-hydroxy-2-phenylethyl]amino]ethyl]phenyl]acetamide) is a β-adrenergic agonist, with high selectivity for β_3_ over other subtypes, which is used as a second line option for treatment of storage symptoms in OAB [14]. Tamsulosin (5-[(2R)-2-[2-(2-ethoxyphenoxy)ethylamino]propyl]-2-methoxybenzenesulfonamide) is an α_1_-adrenoceptor antagonist, with high selectivity for α_1A_ and α_1D_ over α_1B_, and belongs to the most commonly prescribed α_1_-blockers in treatment of voiding symptoms suggestive of BPH [14].

## Figures and Tables

**Table 1:** Close contacts in 10 attempts for docking of mirabegron and 2 for docking of tamsulosin to human α_1A_-adrenergic receptor (PDB ID 7YMH). Contacts shared by all simulations showing binding of mirabegron in slope orientation, shared by all simulations showing mirabegron binding in horizontal orientation, or shared by both simulations with tamsulosin are marked by grey boxes.

	Mirabegron										Tamsulosin	
	#1	#3	#4	#5	#7	#9	#10	#2	#6	#8	#1	#2
Phe-86								+	+	+		+
Trp-102											+	+
Ala-103	+	+	+	+	+	+	+				+	+
Asp-106	+				+						+	+
Val-107	+	+	+	+	+	+	+				+	+
Cys-110				+								
Ser-158	+	+	+	+	+	+	+					
Pro-161	+	+		+	+		+					
Leu-162	+	+	+	+	+	+	+					
Gln-177											+	+
Ile-178	+	+	+	+	+	+	+	+	+		+	+
Asn-179	+	+	+	+	+	+	+					
Glu-180								+	+			
Val-185	+	+	+	+	+	+	+					
Ser-188	+	+	+	+								+
Phe-288	+	+	+	+	+	+	+					
Phe-289	+	+	+	+	+	+	+					
Met-292	+	+	+	+	+	+	+					
Gly-295								+				
Pro-303								+	+	+		
Glu-305										+		
Phe-308								+	+	+		
Lys-309								+	+	+	+	+
Phe-312											+	+
Number of close contacts	13	12	11	13	12	10	11	7	6	5	8	10
Shared contacts	ala-103, val-107, ser-158, leu-162, ile-178, asn-179, val-185, phe-288, phe-289, met-292							phe-86, pro-303, phe-308, lys-309			trp-102, ala-103, asp-106, val-107, gln-177, ile-178, lys-309, phe-312	
Orientation of bound ligand	Slope, slightly vertical							Horizontal, only receptor surface			vertical	

**Table 2:** Close contacts of mirabegron in slope and horizontal binding to an activated α_1A_-adrenoceptor (7YMH) in our simulations (indicated contacts are shared by each simulation showing binding in slope orientation, and by each simulation showing horizontal binding), and interactions of α_1_-adrenergic ligands to cryo-EM structures [18,19] and in homology models (bovine rhodopsin-based homology model [22]; β_2_ homology model and mutagenesis data [25]). A61, A61603; C6, “compound 6” (imidazoline antagonist); ADR, adrenaline; DOX, doxazosin; H, horizontal; M, mirabegron; OXY, oxymetazoline; RWJ, RWJ-69736; S, slope; SIL, silodosin; SN, SNAP-7915; TA, tamsulosin. Note: table continues on next page.

	This Study (7YMH)			Toyoda et al. [18]			Su et al. [19]		Kinsella et al. [22]			Li et al. [25]		
	M/S	M/H	TA	NA	OXY	TA	ADR	A61	TA	DOX	C6	SIL	RWJ	SN
Ser-83						+						+		
Phe-86		+				+								
Glu-87						+								
Trp-102			+			+						+		
Ala-103	+		+											
Asp-106			+	+	+	+	+	+	+	+	+	+	+	+
Val-107	+		+		+	+	+	+				+	+	+
Cys-110				+	+		+	+				+	+	
Thr-111											+	+	+	
Ile-157													+	
Ser-158	+											+		+
Pro-161	(5/7)													+
Leu-162	+													
Arg-166														+
Cys-176						+								
Gln-177			+											
Ile-178	+		+					+	+			+		+
Asn-179	+				+									
Glu-180									+		+			
Tyr-184					+								+	+
Val-185	+				+			+						
Ser-188	(4/7)			+	+	+	+	+			+	+	+	+
Ala-189								+				+		
Ser-192											+	+	+	+
Phe-193											+	+	+	+
Tyr-194											+			
Trp-285				+	+		+	+			+			
Phe-288	+			+	+	+	+	+			+	+	+	+
Phe-289	+			+	+	+	+	+			+	+		+
Met-292	+			+	+	+	+	+		+	+			
Pro-303		+												
Phe-308		+				+					+	+	+	+
Lys-309		+	+									+	+	
Phe-312			+	+	+	+	+	+			+	+	+	+
Trp-313											+			
Gly-315							+	+						
Tyr-316				+	+	+	+	+		+	+	+		

## Data Availability

The original contributions presented in the study are included in the article/Supplementary Materials; further inquiries can be directed to the corresponding author.

## Supplementary Materials

The following supporting information can be downloaded at: https://www.mdpi.com/article/10.3390/ijms25137468/s1.
