# Supplementary material for: Ligand–Receptor Interactions and Structure–Function Relationships in Off-Target Binding of the β3-Adrenergic Agonist Mirabegron to α1A-Adrenergic Receptors
Source: Int J Mol Sci. 2024 Jul 7;25(13):7468. doi: 10.3390/ijms25137468 (PMC11242030; doi:10.3390/ijms25137468)

Mirabegron, #1 (slope)

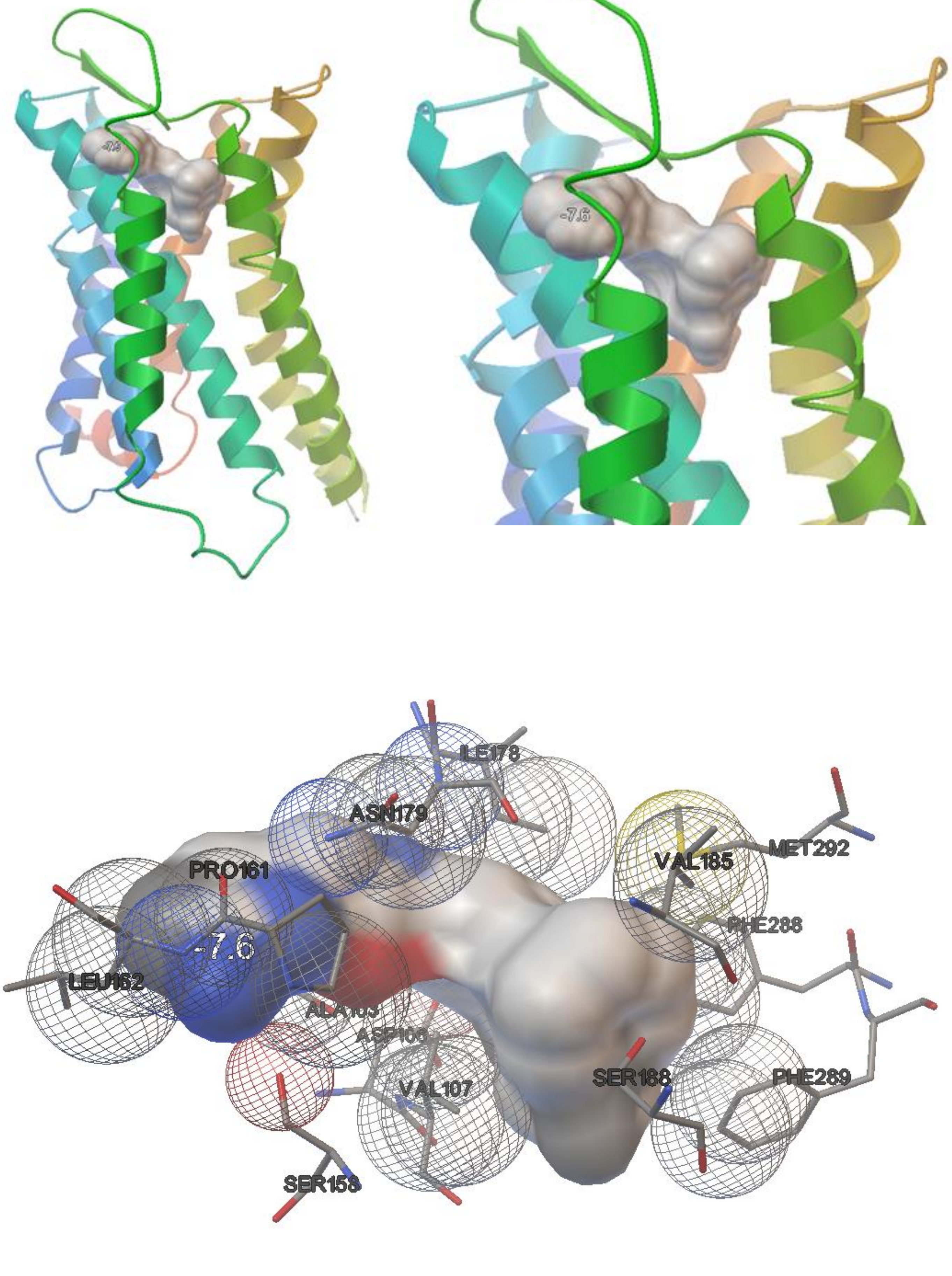

Mirabegron, #3 (slope)

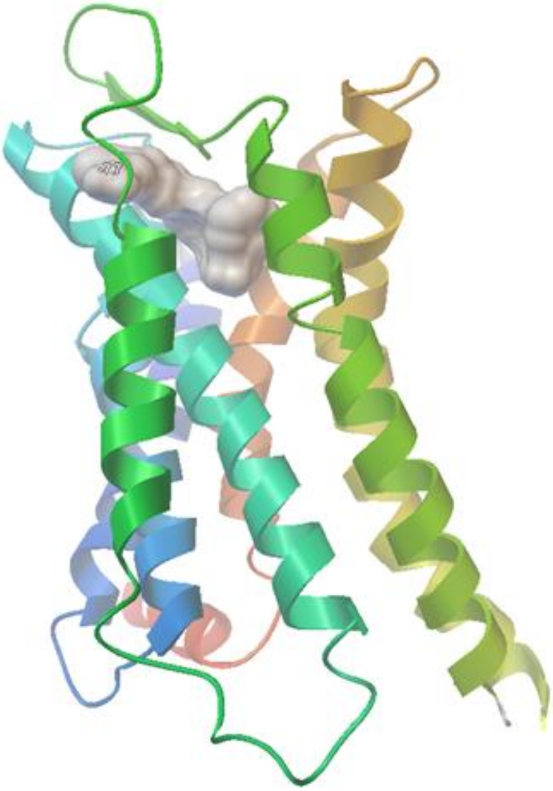

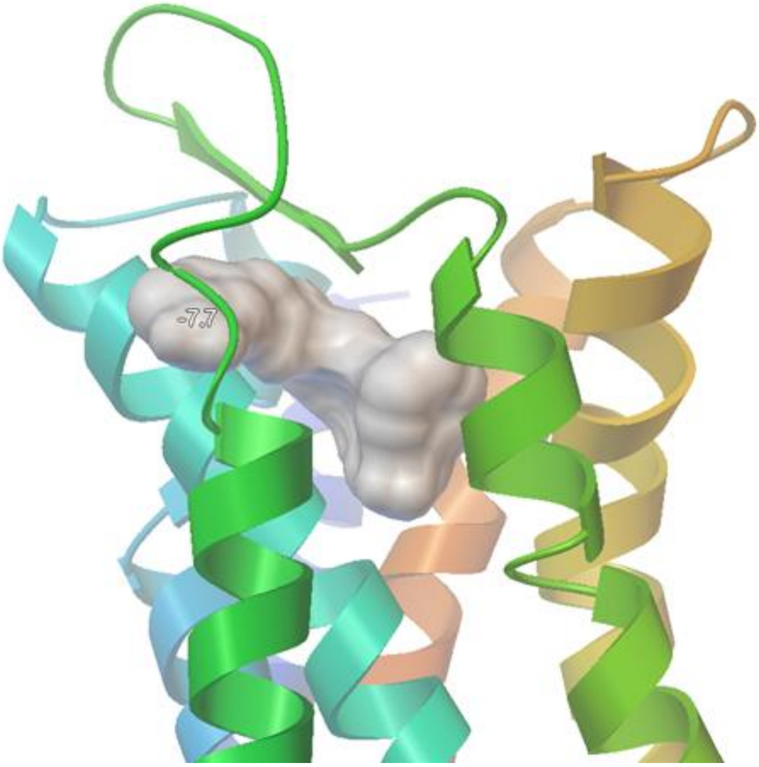

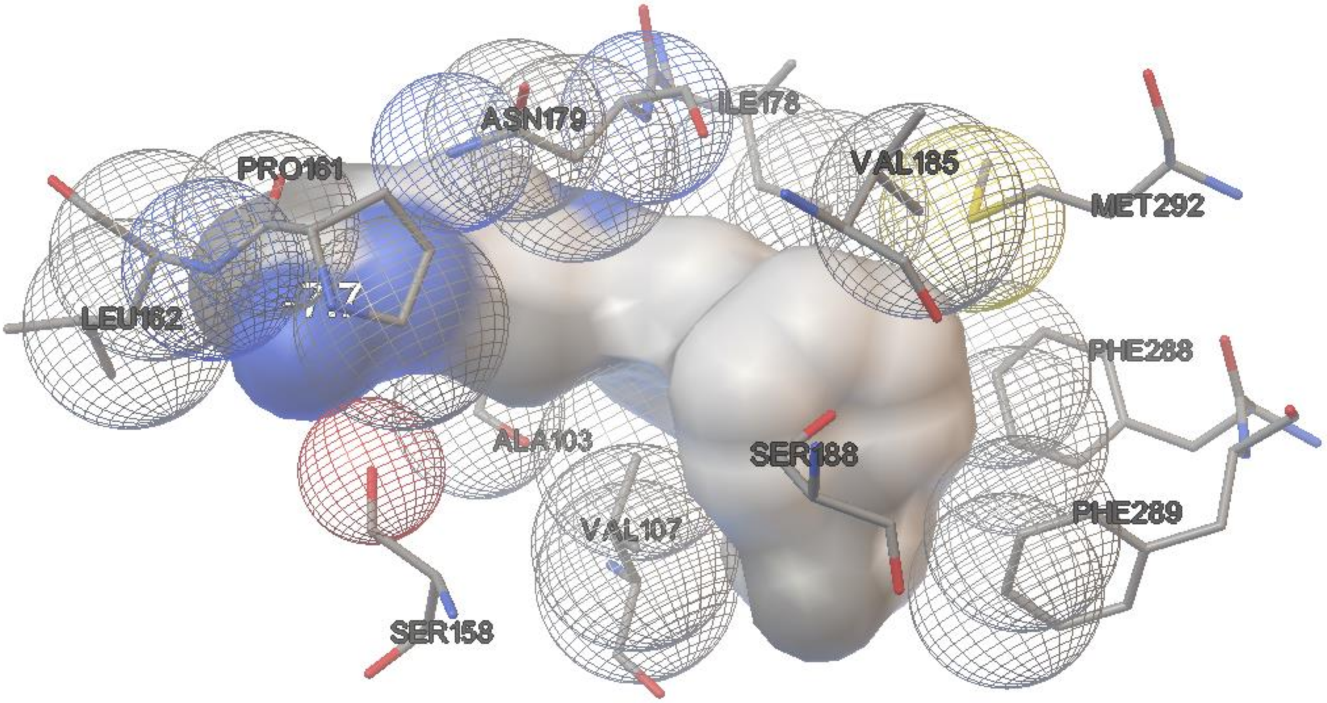

Mirabegron, #4 (slope)

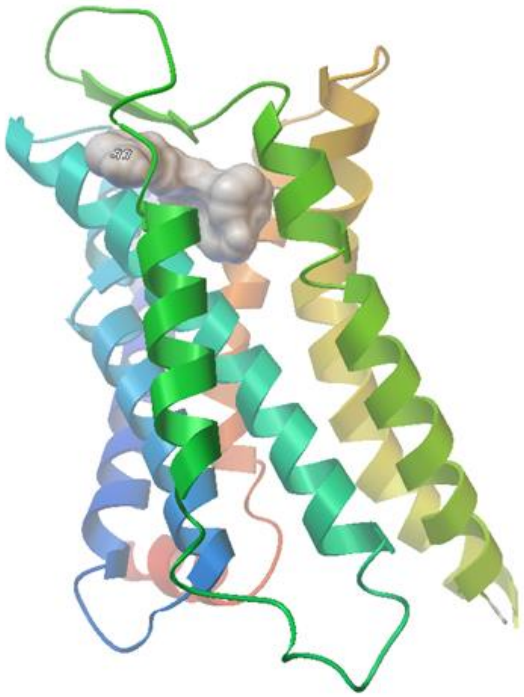

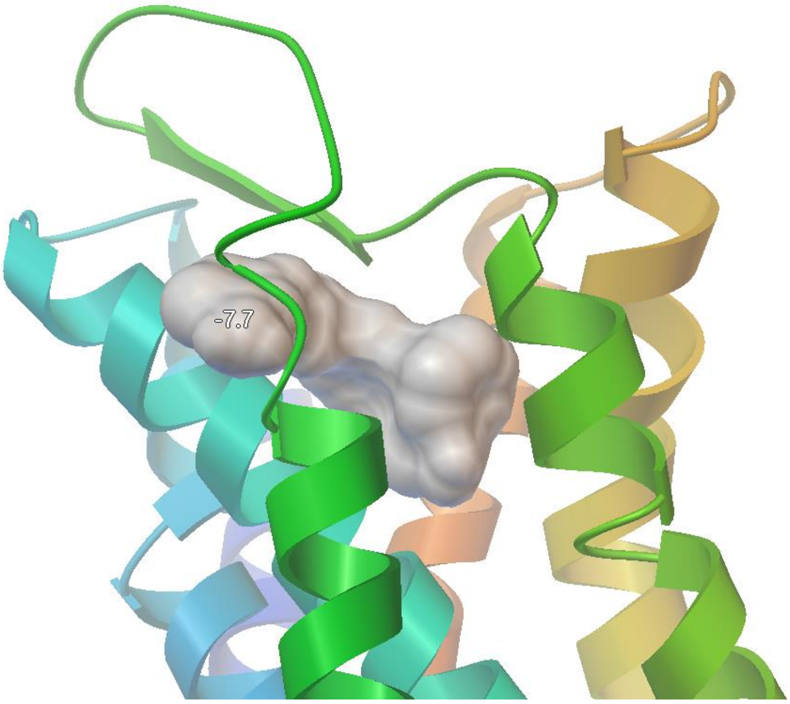

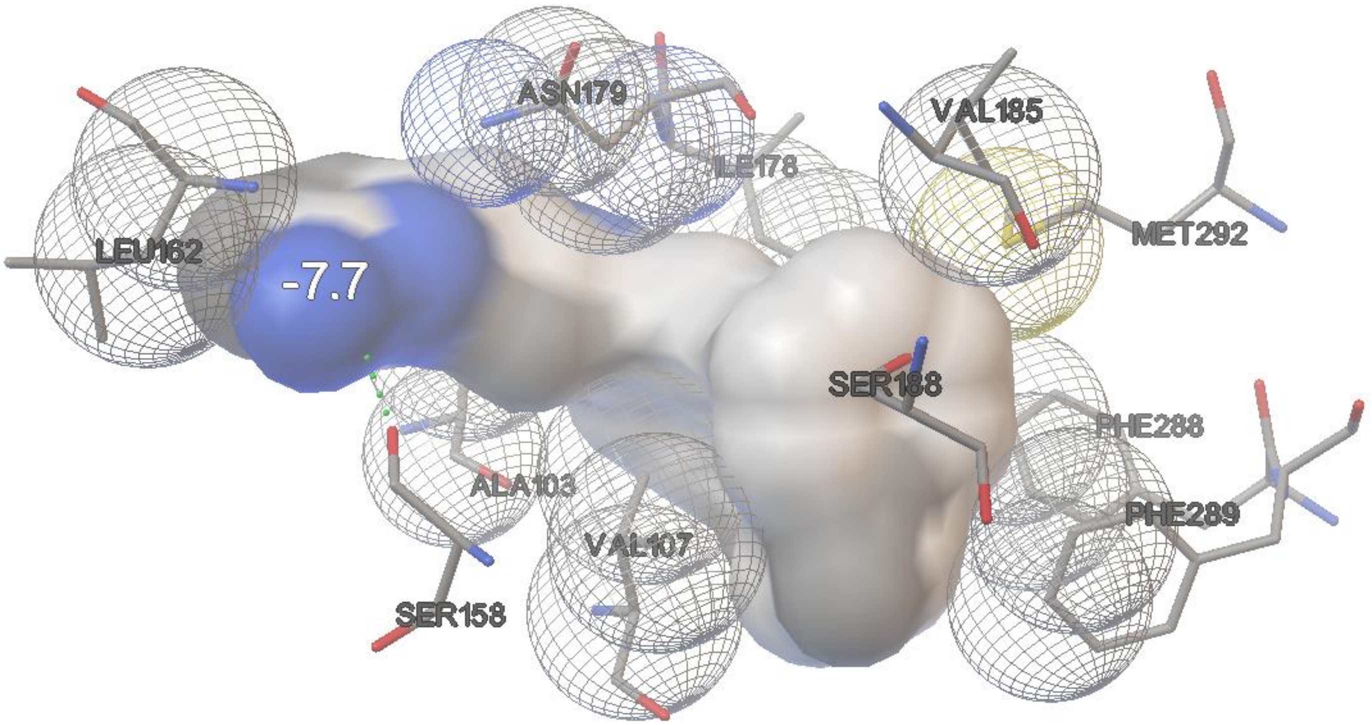

Mirabegron, #5 (slope)

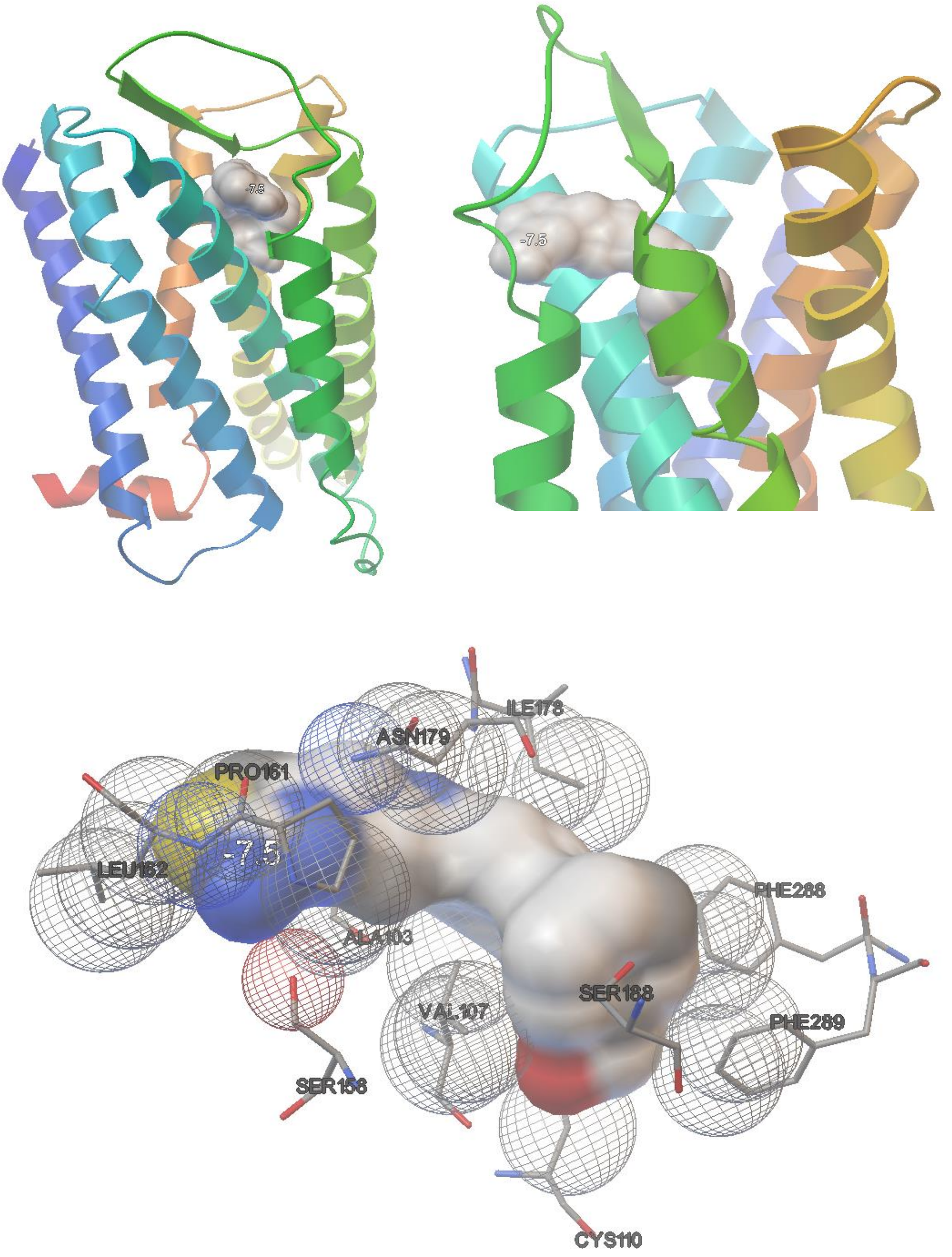

Mirabegron, #7 (slope)

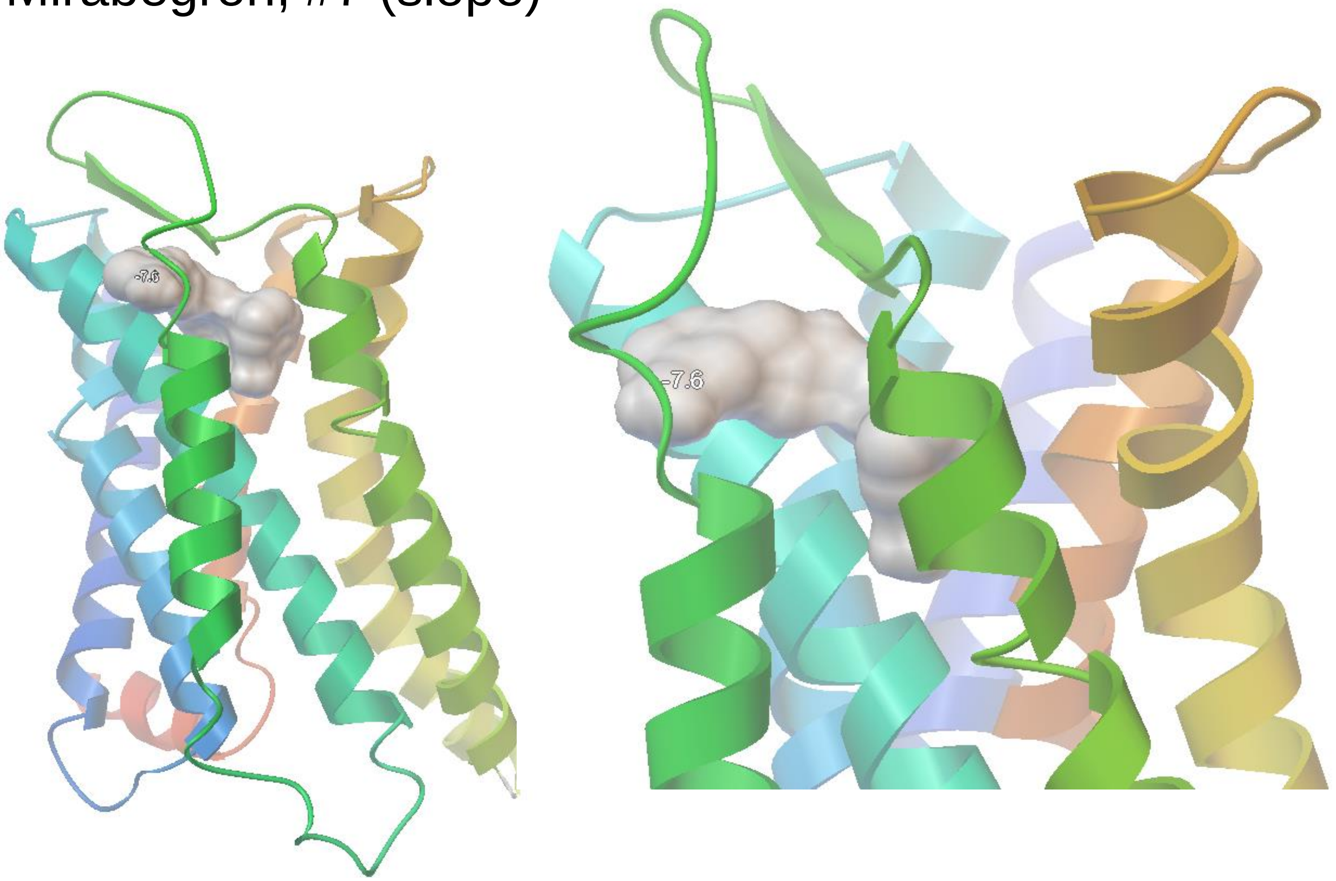

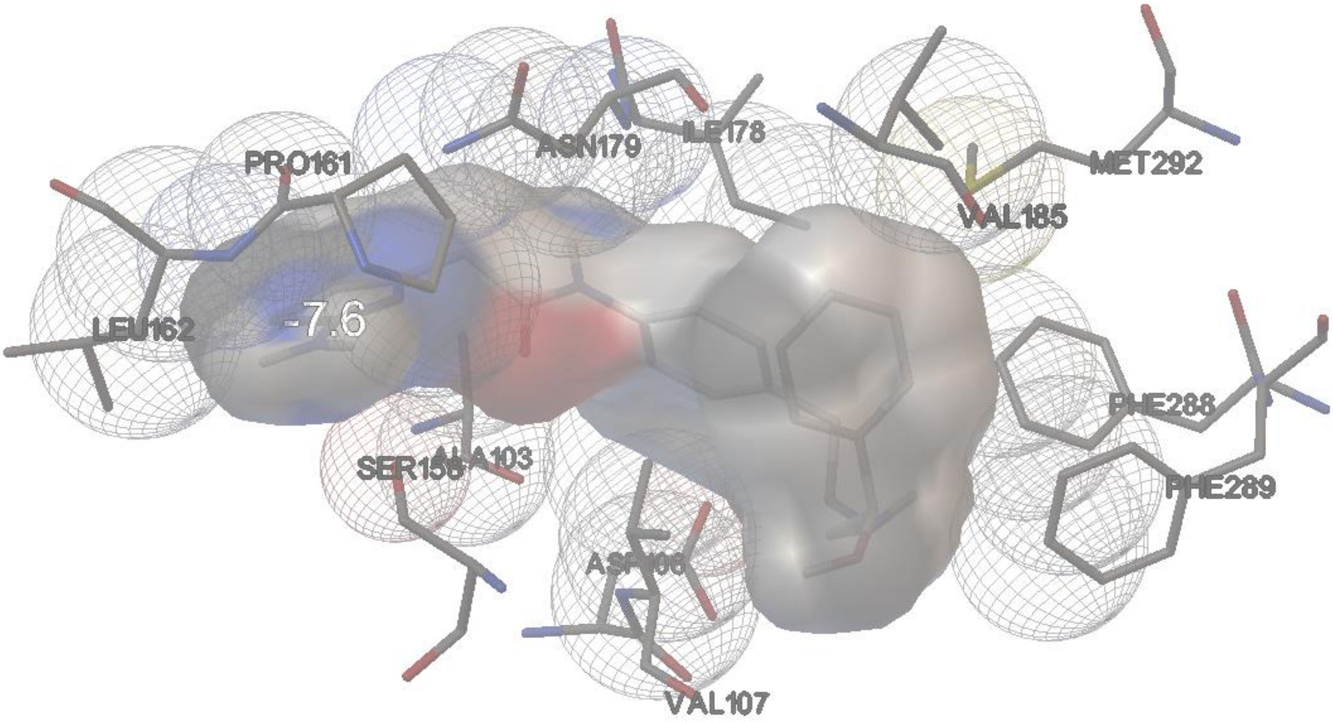

Mirabegron, #9 (slope)

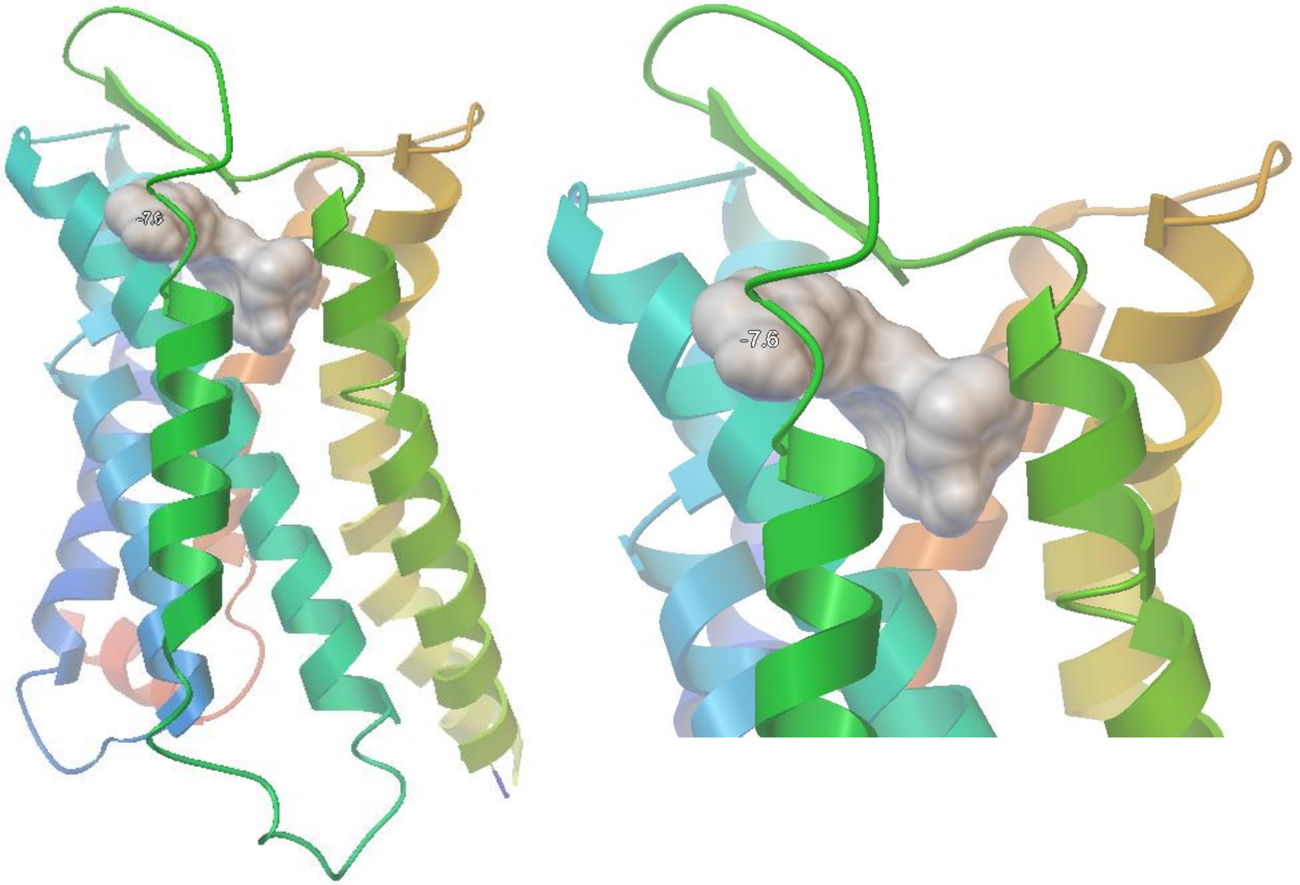

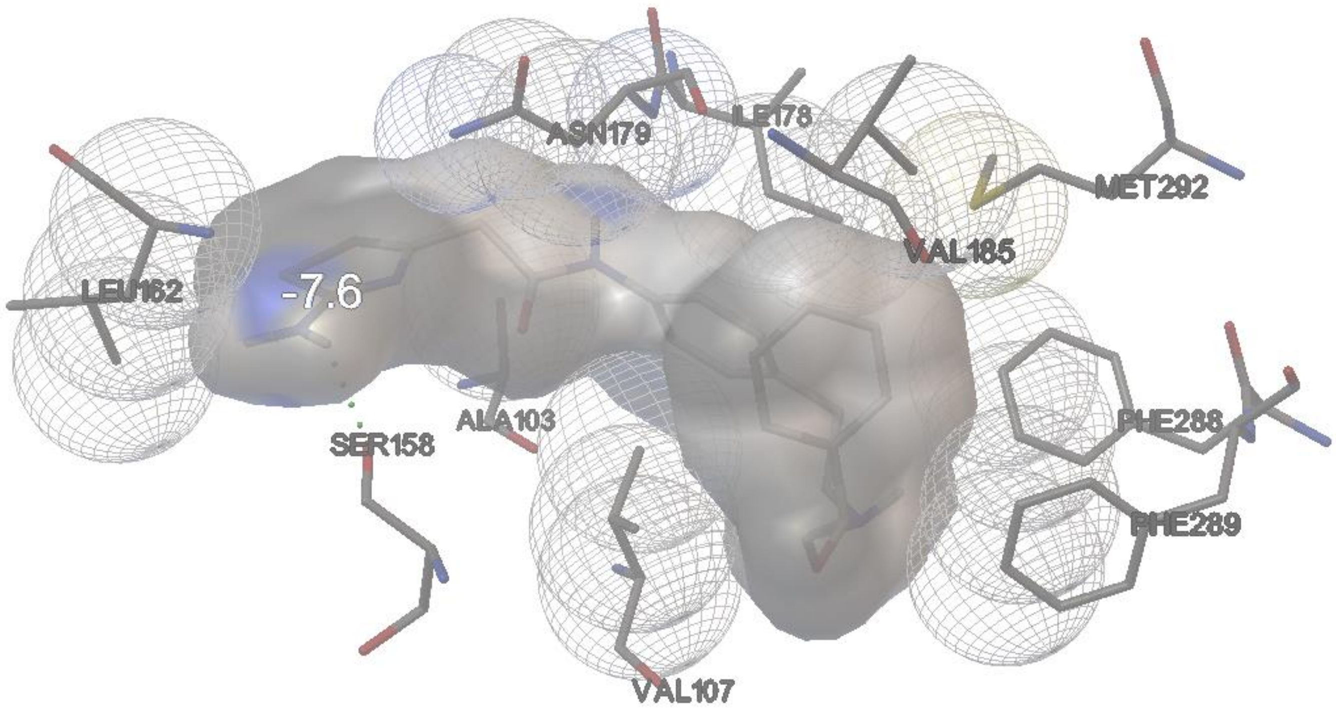

Mirabegron, #10 (slope)

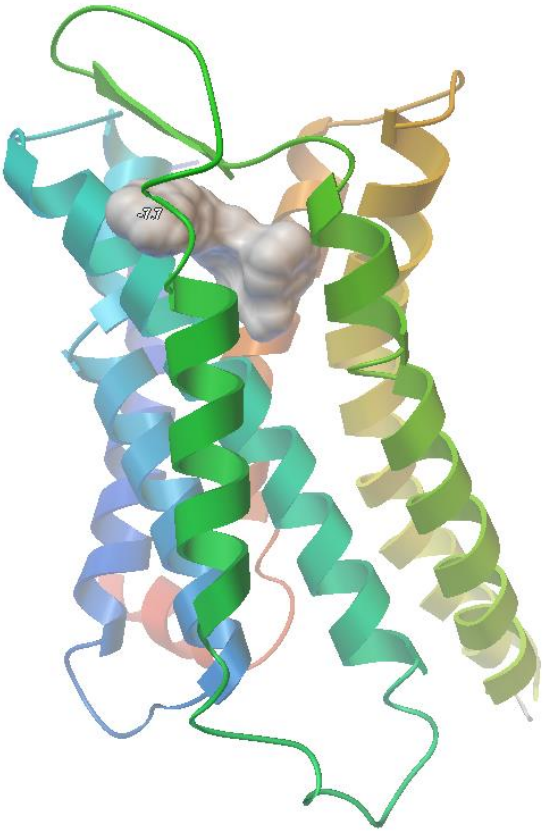

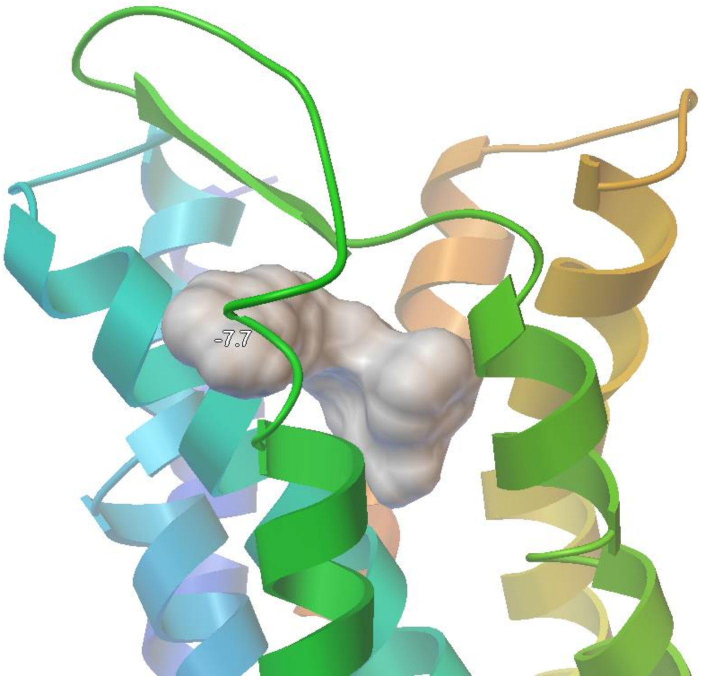

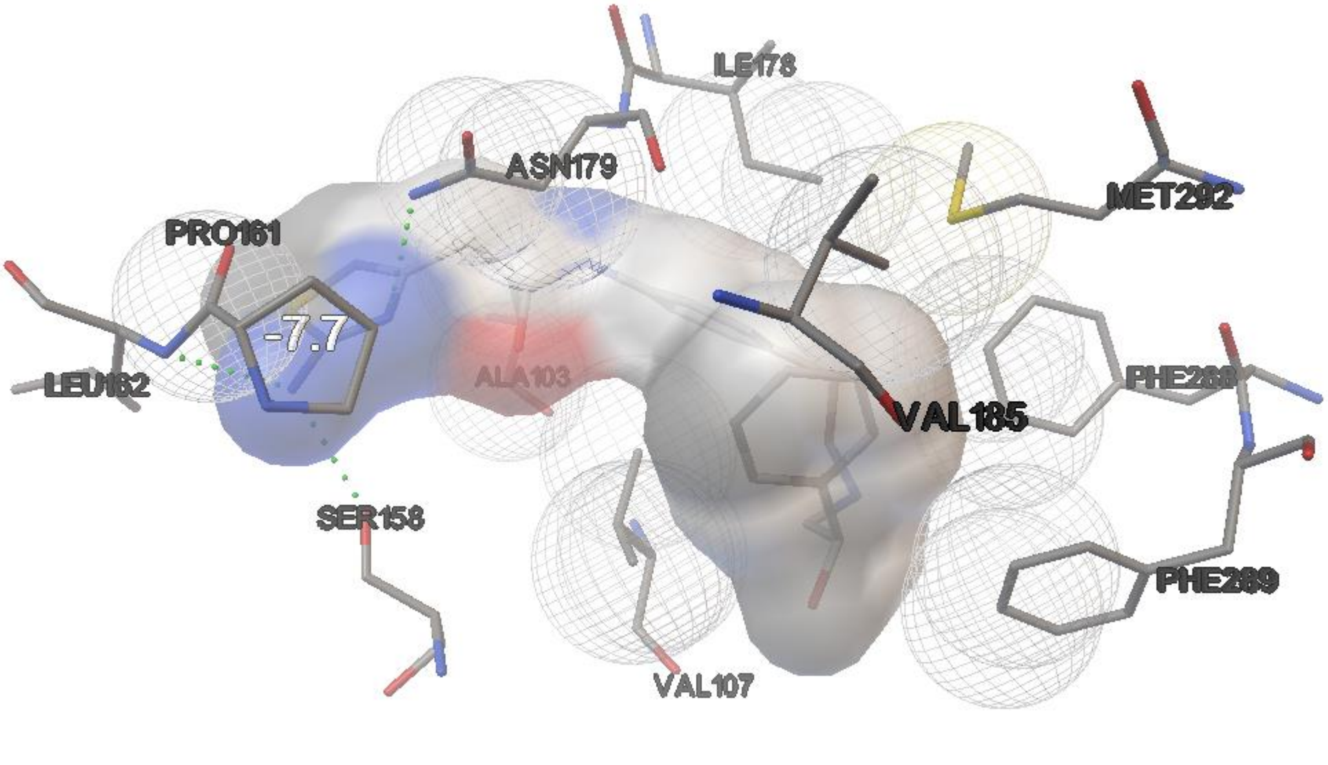

Mirabegron, #2 (horizontal)

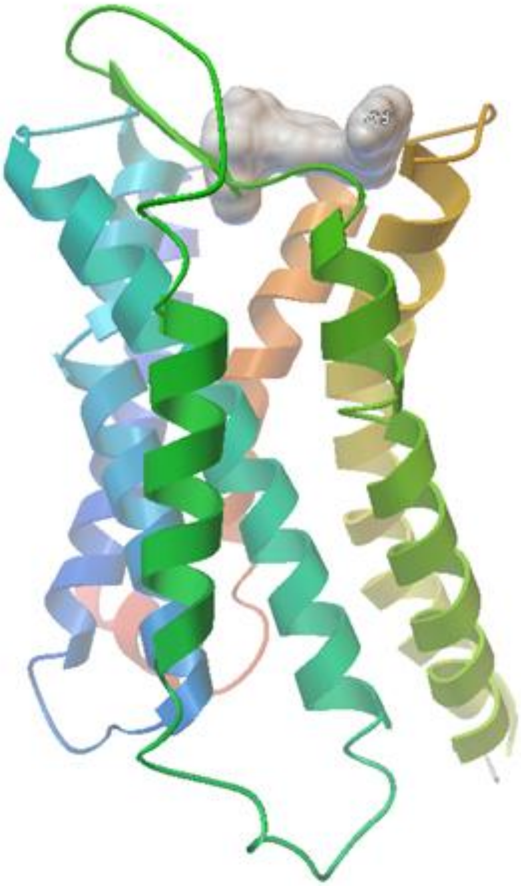

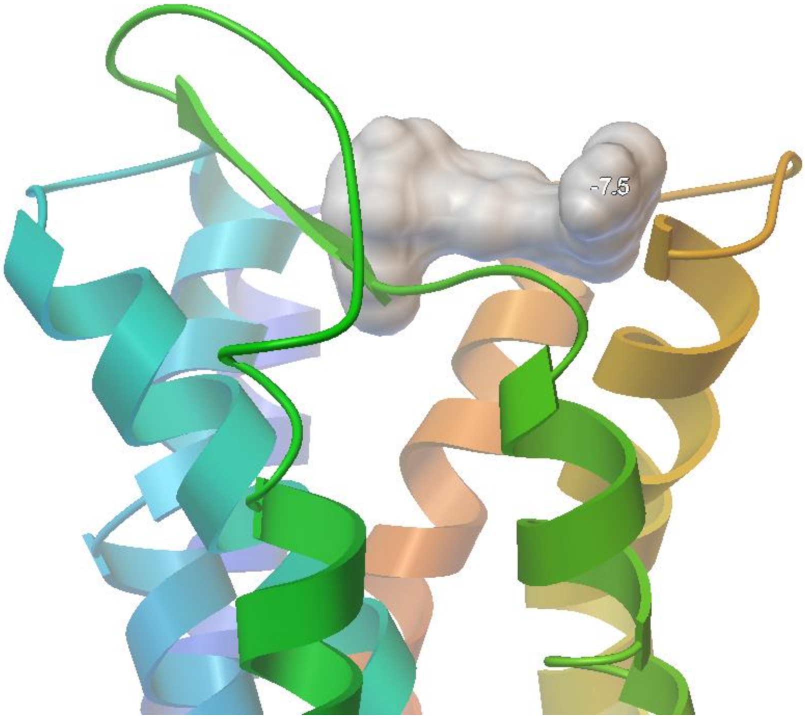

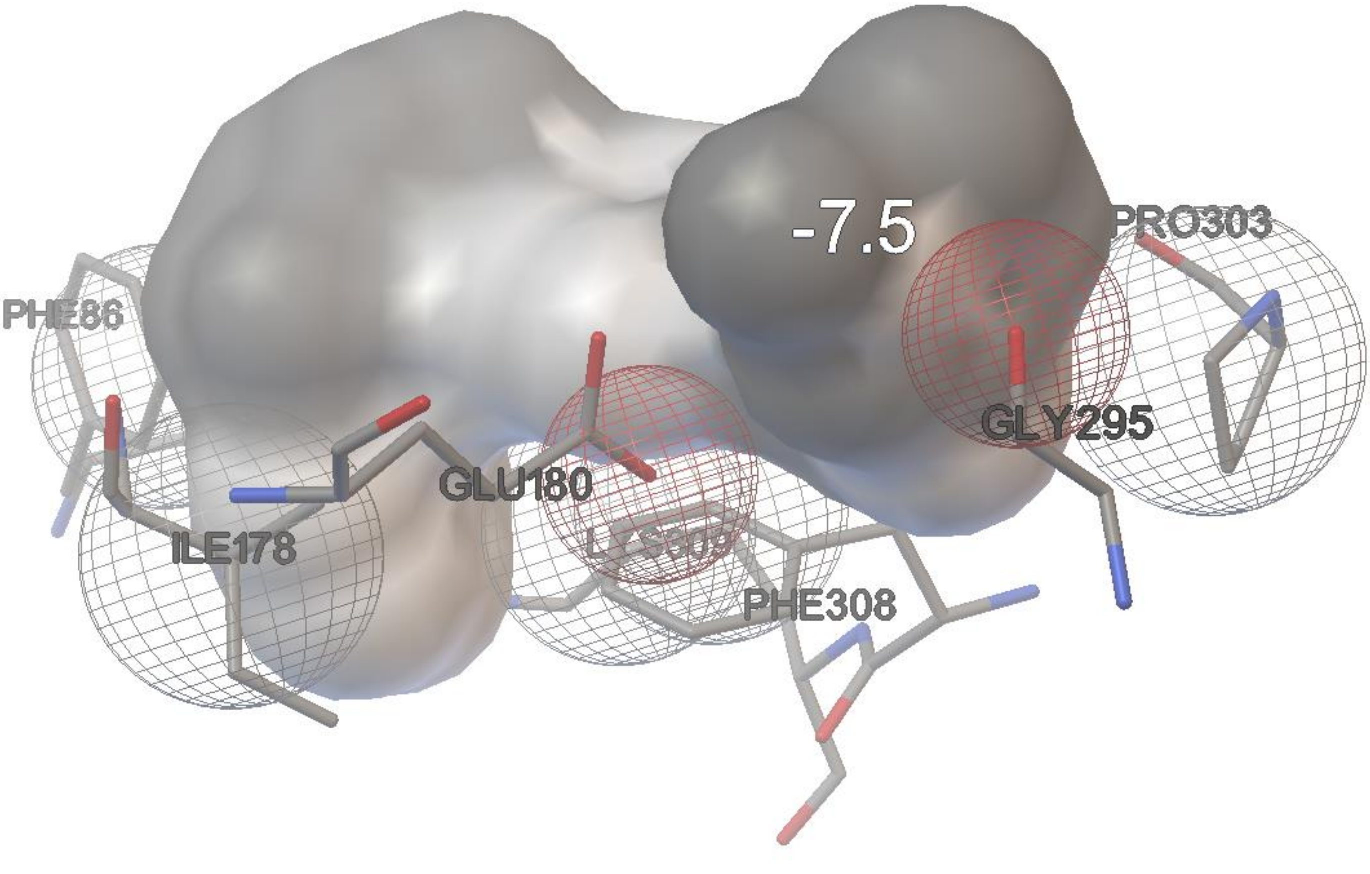

Mirabegron, #6 (horizontal)

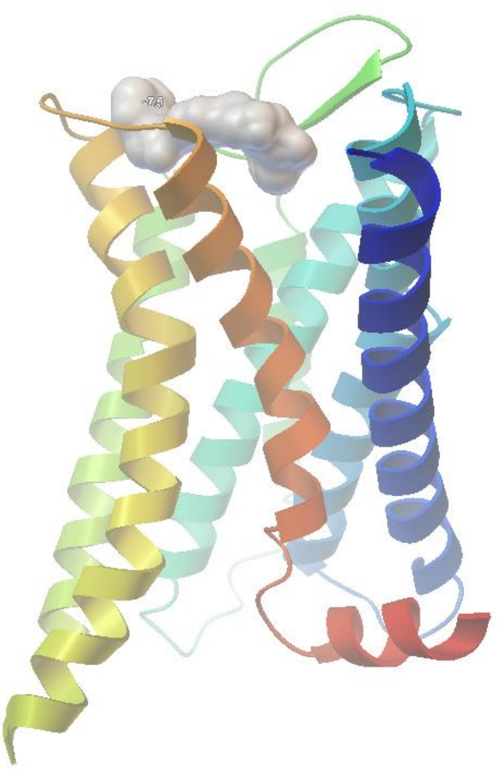

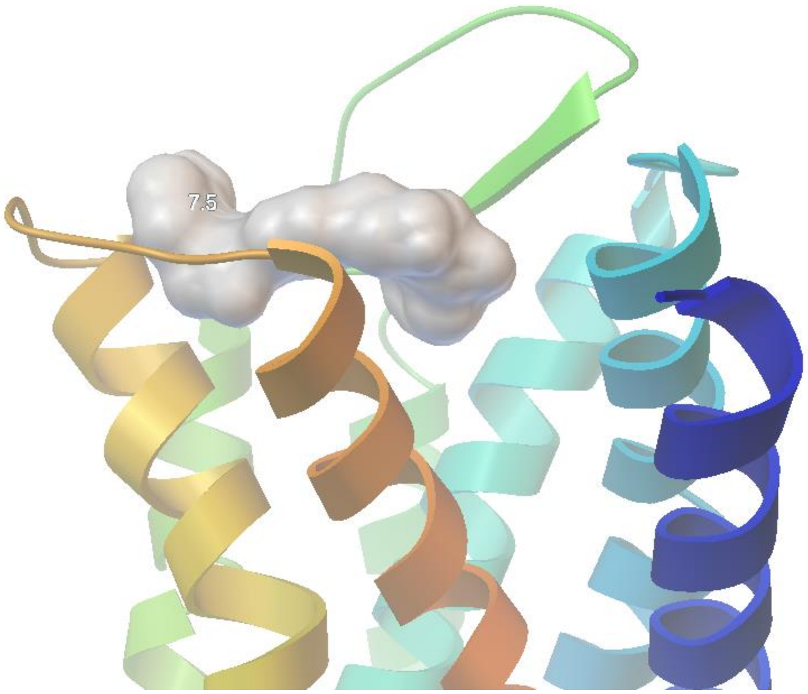

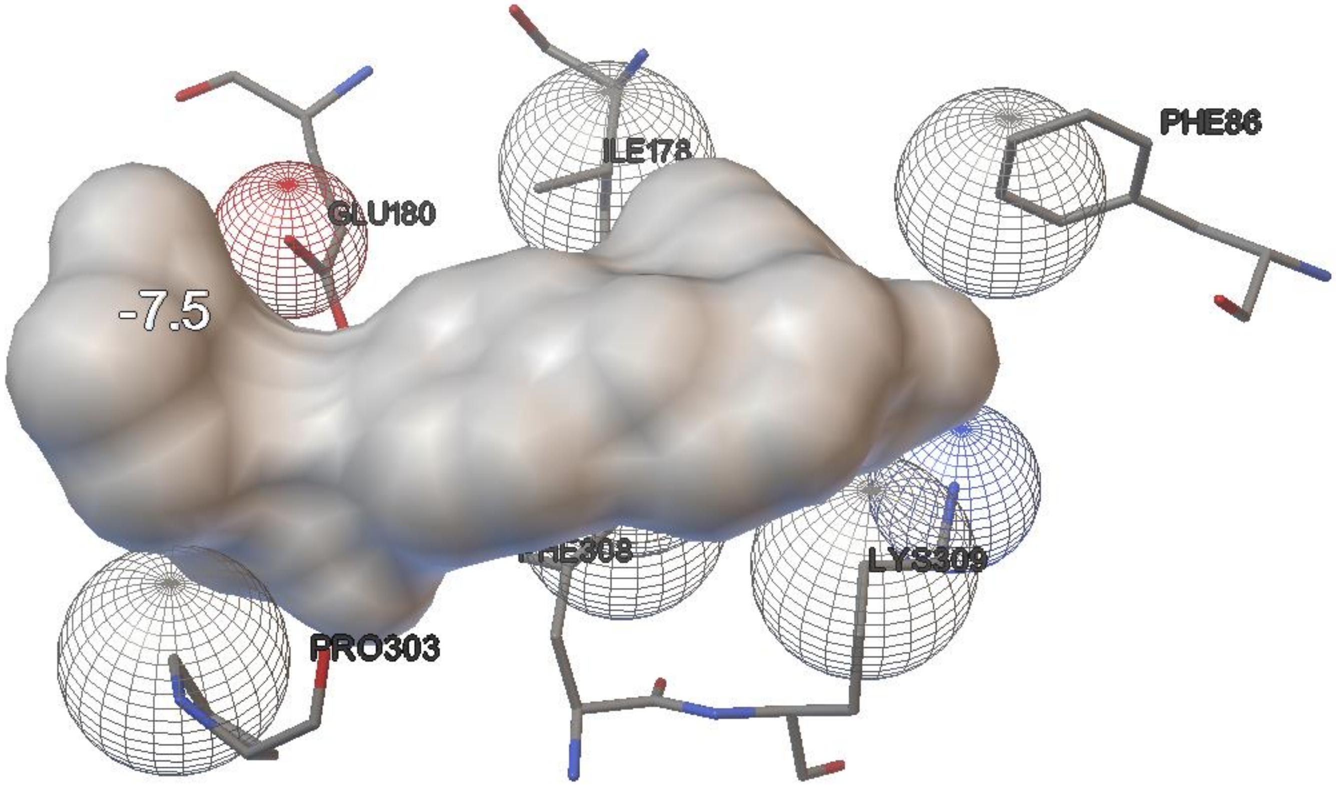

Mirabegron, #8 (horizontal)

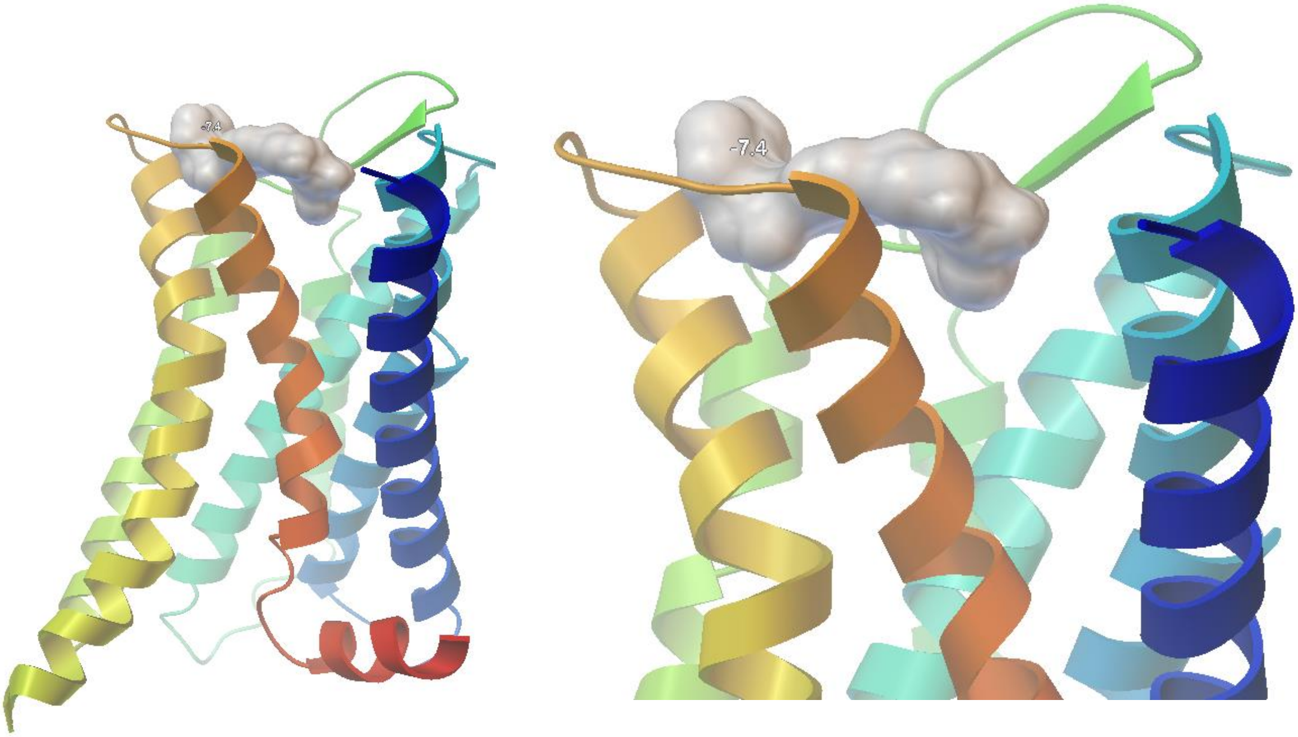

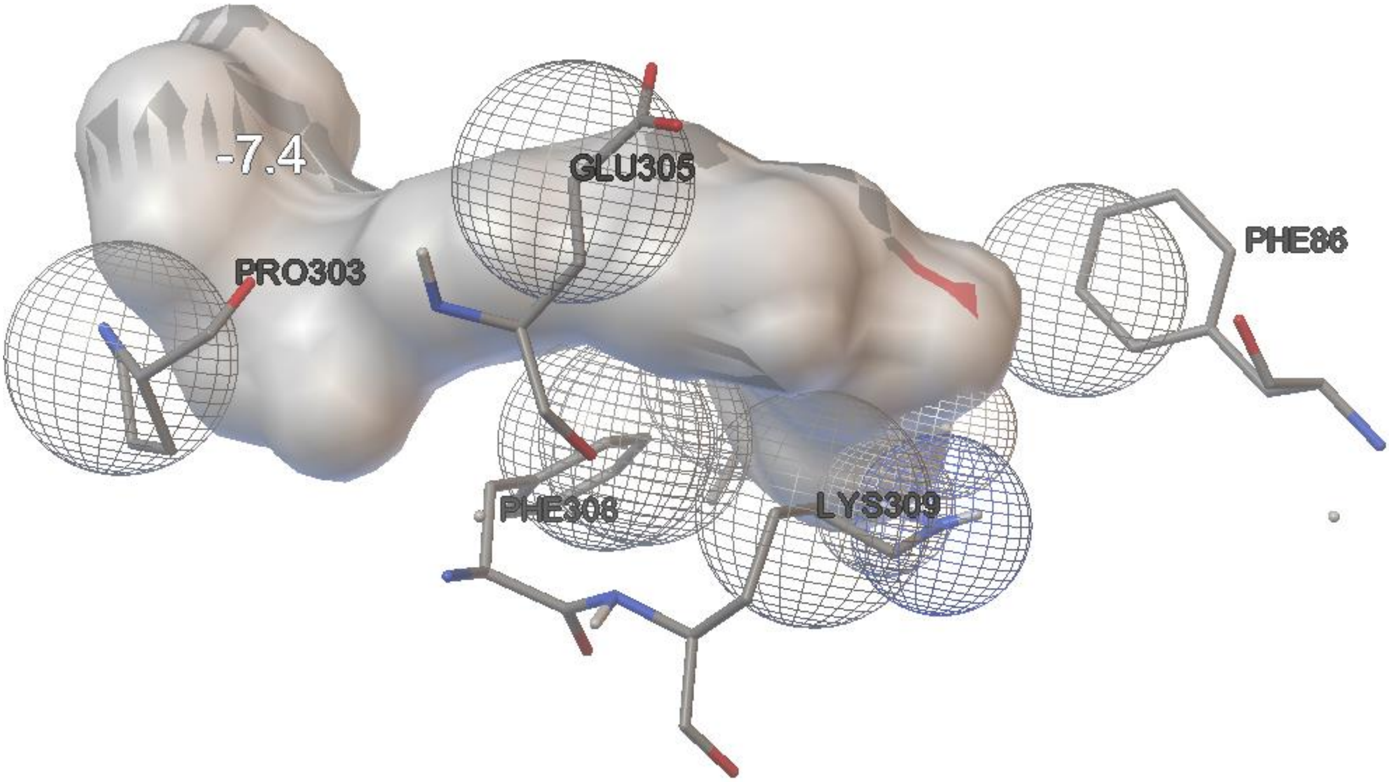

Tamsulosin, #1

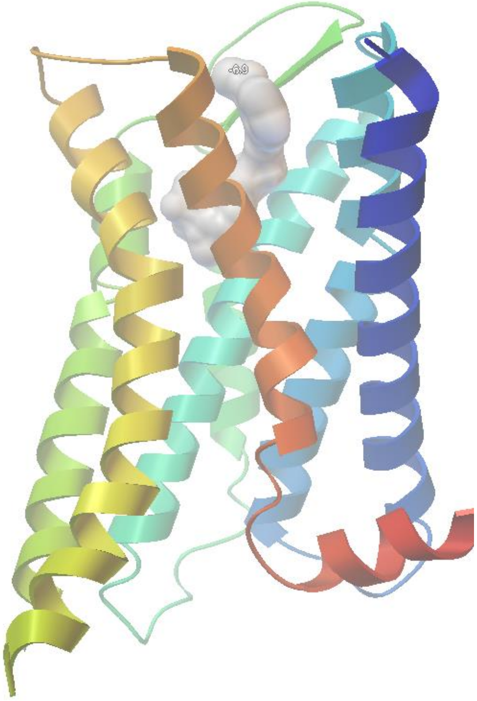

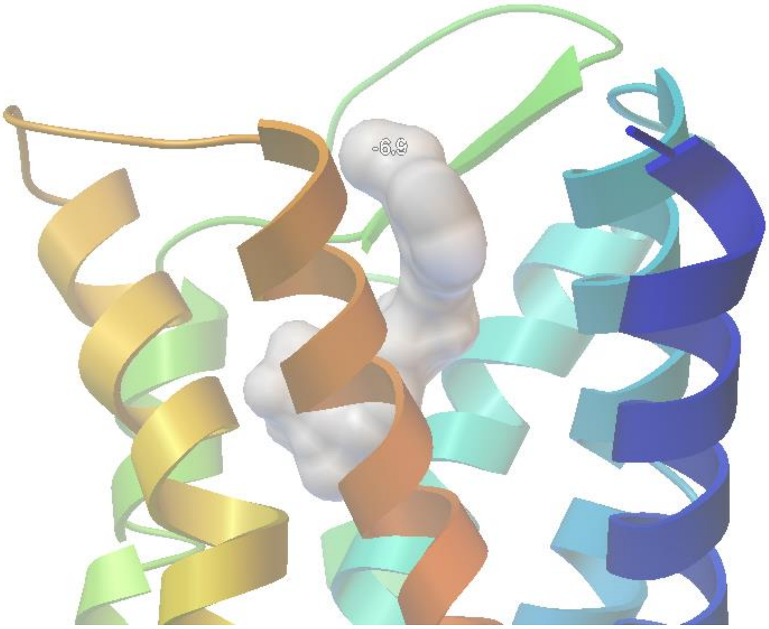

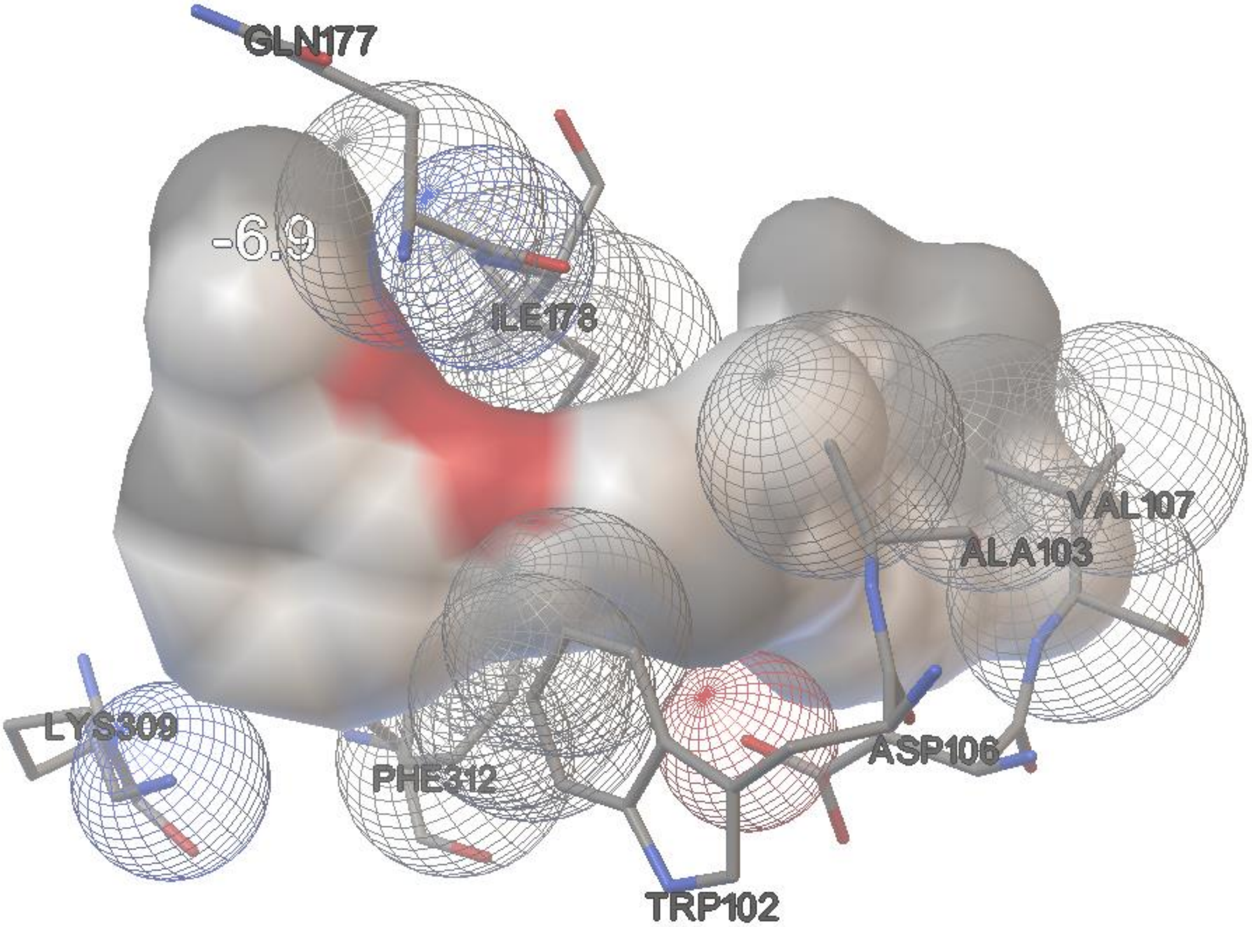

Tamsulosin, #2

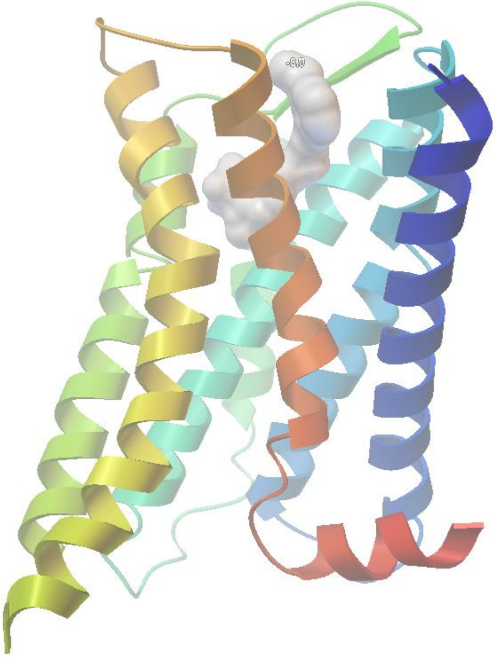

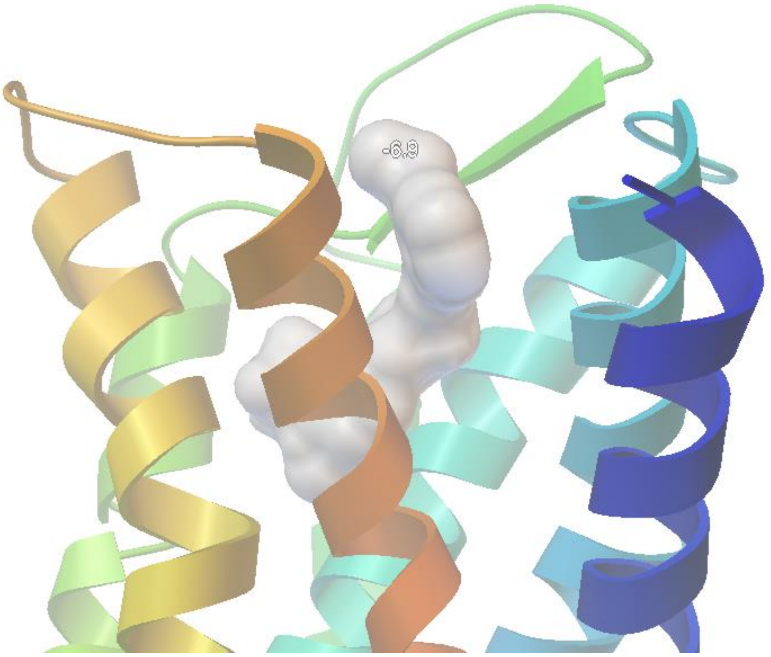

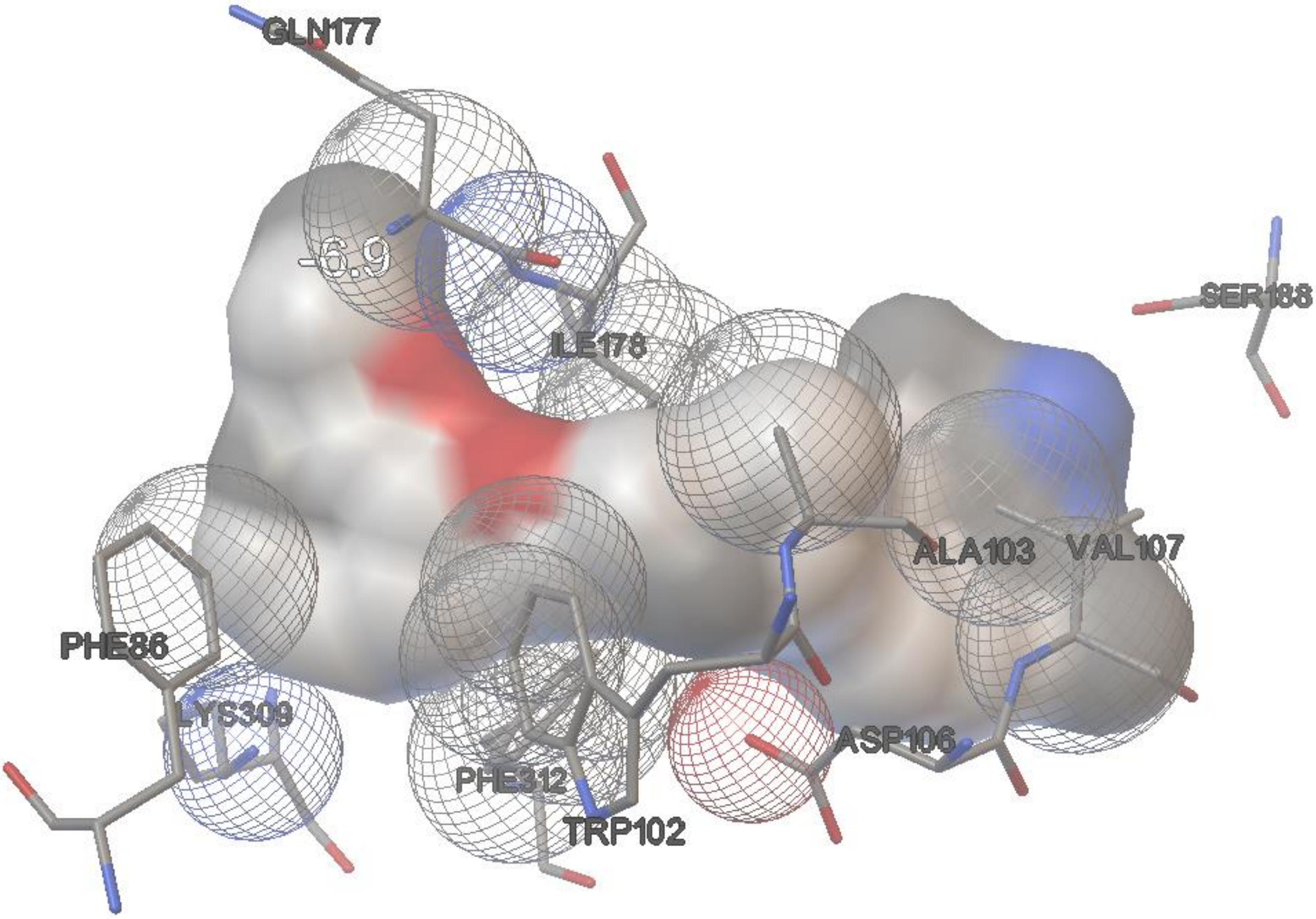

Supplement: Supplementary file 1 [file ijms-25-07468-s001.zip › ijms-3076210-supplementary.pdf]
